# Supplementary material for: Assignment of protonated R-homocitrate in extracted FeMo-cofactor of nitrogenase via vibrational circular dichroism spectroscopy
Source: Commun Chem. 2020 Oct 28;3:145. doi: 10.1038/s42004-020-00392-z (PMC8323615; doi:10.1038/s42004-020-00392-z)
Supplement: Supplementary file 1 — Supplementary Information [file 42004_2020_392_MOESM1_ESM.pdf]

Supporting materials

Assignment of protonated *R*-homocitrate in extracted FeMo-cofactor of nitrogenase *via* vibrational circular dichroism spectroscopy

Lan Deng,[1] Hongxin Wang,[2] Christie H. Dapper,[3] William E. Newton,[3] Sergey Shilov,[4] Shunlin Wang,[4] Stephen P. Cramer[2]* and Zhao-Hui Zhou[1]*

[1]*State Key Laboratory of Physical Chemistry of Solid Surfaces and Department of Chemistry, College of Chemistry and Chemical Engineering, Xiamen University, Xiamen, 361005, China*

[2]*SETI Institute, Mountain View, CA 94043, USA*

[3]*Department of Biochemistry, Virginia Polytechnic Institute and State University, Blacksburg, VA 24061, USA*

[4]*Bruker Optics, 19 Fortune Dr., Billerica, MA 01821, USA*

Figure and Table Options

TABLE OF CONTENTS

Supplementary Methods

Part I Syntheses, spectral characterizations and structural analyses for molybdenum *α*-hydroxycarboxylates

More physical measurements, preparations of [Λ/Δ-$Mo^*_2O_2(\mu_2$-S)(μ_2-O)(*S*-$Hlact^*)_2(trz)_2$(trz)] (**2**), [$Mo_2O_2(\mu_2$-S)(μ_2-O)(Hglyc)$_2$(trz)$_2$(H_2O)] (**3**), {Na_2[Δ-Mo^*O_2(*R*-$lact^*)_2$]}$_3$·13H_2O and K_5[Λ,Λ,Λ,Λ-$Mo^*_4O_{11}$(*R*-$Hhomocit^*)_2$]Cl·5H_2O (**21**), gas adsorption and X-ray crystallography.

Supplementary Fig. 1. Syntheses and possible transformations of dinuclear oxidomolybdenum(V) triazole lactates **1**, **2** and glycolates **3** respectively.
Supplementary Fig. 2. Molecular structures of [Δ/Λ-$Mo^*_2O_2(\mu_2$-S)(μ_2-O)(*R*-$Hlact^*)_2(trz)_2$(trz)]·½H_2O (**1**, a) and its enantiomer [Λ/Δ-$Mo^*_2O_2(\mu_2$-S)(μ_2-O)(*S*-$Hlact^*)_2(trz)_2$(trz)] (**2**, b), free water molecule in **1** was omitted for clarity.
Supplementary Fig. 3. Molecular structure of [$Mo_2O_2(\mu_2$-S)(μ_2-O)(Hglyc)$_2$(trz)$_2$(H_2O)] (**3**).
Supplementary Fig. 4. 2D layered diagrams in [Δ/Λ-$Mo^*_2O_2(\mu_2$-S)(μ_2-O)(*R*-$Hlact^*)_2(trz)_2$(trz)]·½H_2O (**1**).
Supplementary Fig. 5. 2D layered diagrams in [Λ/Δ-$Mo^*_2O_2(\mu_2$-S)(μ_2-O)(*S*-$Hlact^*)_2(trz)_2$(trz)] (**2**).
Supplementary Fig. 6. 2D layered diagrams in [$Mo_2O_2(\mu_2$-S)(μ_2-O)(Hglyc)$_2$(trz)$_2$(H_2O)] (**3**).
Supplementary Fig. 7. The channels existed in [Λ/Δ-$Mo^*_2O_2(\mu_2$-S)(μ_2-O)(*S*-$Hlact^*)_2(trz)_2$(trz)] (**2**) viewed along *a* axis.
Supplementary Fig. 8. (a) Adsorption isotherms of **2** for the gases of O_2, CO_2, CH_4, N_2 and H_2 at 298 K at different pressures. Independent O_2 (b), CO_2 (c), CH_4 (d), N_2 (e) and H_2 (f) adsorption isotherms of [Λ/Δ-$Mo^*_2O_2(\mu_2$-S)(μ_2-O)(*S*-$Hlact^*)_2(trz)_2$(trz)] (**2**) at 298 K in different pressures.
Supplementary Fig. 9. Independent O_2 (a) and N_2 (b) adsorption isotherms of [Δ/Λ-$Mo^*_2O_2(\mu_2$-S)(μ_2-O)(*R*-$Hlact^*)_2(trz)_2$(trz)]·½H_2O (**1**) at 298 K in different pressures.
Supplementary Fig. 10. VCD and IR spectra of {Na_2[Δ-Mo^*O_2(*R*-$lact^*)_2$]}$_3$·13H_2O (a) and {Na_2[Λ-Mo^*O_2(*S*-$lact^*)_2$]}$_3$·13H_2O (b) in the regions of 1800 − 800 cm^{-1}.
Supplementary Fig. 11. VCD and IR spectra of [Δ/Λ-$Mo^*_2O_2(\mu_2$-S)(μ_2-O)(*R*-$Hlact^*)_2(trz)_2$(trz)]·½H_2O (**1**) and

{Na_2[Δ-Mo^*O_2(*R*-$lact^*)_2$]}$_3$·13H_2O using KBr pellet in the regions of 1800 – 800 cm^{-1}.

Supplementary Fig. 12. Comparisons of VCD and IR spectra of [Λ/Δ-$Mo^*_2O_2$(μ_2-S)(μ_2-O)(*S*-$Hlact^*)_2$(trz)$_2$(trz)] (**2**, a) and {Na_2[Λ-Mo^*O_2(*S*-$lact^*)_2$]}$_3$·13H_2O (b) using KBr pellet in the regions of 1800 – 800 cm^{-1}.

Supplementary Fig. 13. Optimized molecular structure of [Δ/Λ-$Mo^*_2O_2$(μ_2-S)(μ_2-O)(*R*-$Hlact^*)_2$(trz)$_2$(trz)]·½H_2O (**1**). Color: molybdenum, violet; oxygen, red; nitrogen, blue; carbon, gray; sulfur, yellow; hydrogen, white.

Supplementary Fig. 14. Optimized molecular structure of Δ-$Mo^*Fe_7S_9C$[*R*-(H)$homocit^*$](NMF)$_2$ (**20**). Color: molybdenum, violet; oxygen, red; nitrogen, blue; carbon, gray; sulfur, yellow; hydrogen, white; iron, slate.

Supplementary Fig. 15. IR spectra of [Δ/Λ-$Mo^*_2O_2$(μ_2-S)(μ_2-O)(*R*-$Hlact^*)_2$(trz)$_2$(trz)]·½H_2O (**1**), [Λ/Δ-$Mo^*_2O_2$(μ_2-S)(μ_2-O)(*S*-$Hlact^*)_2$(trz)$_2$(trz)] (**2**), and [Mo_2O_2(μ_2-S)(μ_2-O)(Hglyc)$_2$(trz)$_2$(H_2O)] (**3**), in the region of 4000 ~ 450 cm^{-1}.

Supplementary Fig. 16. IR spectra of [Δ/Λ-$Mo^*_2O_2$(μ_2-S)(μ_2-O)(*R*-$Hlact^*)_2$(trz)$_2$(trz)]·½H_2O (**1**), [Λ/Δ-$Mo^*_2O_2$(μ_2-S)(μ_2-O)(*S*-$Hlact^*)_2$(trz)$_2$(trz)] (**2**) and [Mo_2O_2(μ_2-S)(μ_2-O)(Hglyc)$_2$(trz)$_2$(H_2O)] (**3**), in the region of 600 ~ 460 cm^{-1}.

Supplementary Fig. 17. Solid diffused reflectance UV-Vis spectra of [Δ/Λ-$Mo^*_2O_2$(μ_2-S)(μ_2-O)(*R*-$Hlact^*)_2$(trz)$_2$(trz)]·½H_2O (**1**), [Λ/Δ-$Mo^*_2O_2$(μ_2-S)(μ_2-O)(*S*-$Hlact^*)_2$(trz)$_2$(trz)] (**2**) and [Mo_2O_2(μ_2-S)(μ_2-O)(Hglyc)$_2$(trz)$_2$(H_2O)] (**3**).

Supplementary Fig. 18. Solid-state ^1H NMR spectra of [Δ/Λ-$Mo^*_2O_2$(μ_2-S)(μ_2-O)(*R*-$Hlact^*)_2$(trz)$_2$(trz)]·½H_2O (**1**, a), [Λ/Δ-$Mo^*_2O_2$(μ_2-S)(μ_2-O)(*S*-$Hlact^*)_2$(trz)$_2$(trz)] (**2**, b) and [Mo_2O_2(μ_2-S)(μ_2-O)(Hglyc)$_2$(trz)$_2$(H_2O)] (**3**, c).

Supplementary Fig. 19. Solid-state ^{13}C NMR spectra of [Δ/Λ-$Mo^*_2O_2$(μ_2-S)(μ_2-O)(*R*-$Hlact^*)_2$(trz)$_2$(trz)]·½H_2O (**1**, a), [Λ/Δ-$Mo^*_2O_2$(μ_2-S)(μ_2-O)(*S*-$Hlact^*)_2$(trz)$_2$(trz)] (**2**, b) and [Mo_2O_2(μ_2-S)(μ_2-O)(Hglyc)$_2$(trz)$_2$(H_2O)] (**3**, c).

Supplementary Fig. 20a. TG–DTG curves of [Δ/Λ-$Mo^*_2O_2$(μ_2-S)(μ_2-O)(*R*-$Hlact^*)_2$(trz)$_2$(trz)]·½H_2O (**1**).

Supplementary Fig. 20b. TG–DTG curves of [Λ/Δ-$Mo^*_2O_2$(μ_2-S)(μ_2-O)(*S*-$Hlact^*)_2$(trz)$_2$(trz)] (**2**).

Supplementary Fig. 20c. TG–DTG curves of [Mo_2O_2(μ_2-S)(μ_2-O)(Hglyc)$_2$(trz)$_2$(H_2O)] (**3**).

Supplementary Fig. 21. TG–DTA curves of [Δ/Λ-$Mo^*_2O_2$(μ_2-S)(μ_2-O)(*R*-$Hlact^*)_2$(trz)$_2$(trz)]·½H_2O (**1**, a), [Λ/Δ-$Mo^*_2O_2$(μ_2-S)(μ_2-O)(*S*-$Hlact^*)_2$(trz)$_2$(trz)] (**2**, b) and [Mo_2O_2(μ_2-S)(μ_2-O)(Hglyc)$_2$(trz)$_2$(H_2O)] (**3**, c).

Supplementary Fig. 22. X-bands of EPR spectra for [Δ/Λ-$Mo^*_2O_2$(μ_2-S)(μ_2-O)(*R*-$Hlact^*)_2$(trz)$_2$(trz)]·½H_2O (**1**),

[Λ/Δ-Mo$^*_2$O$_2$(μ$_2$-S)(μ$_2$-O)(*S*-Hlact*)$_2$(trz)$_2$(trz)] (**2**) and [Mo$_2$O$_2$(μ$_2$-S)(μ$_2$-O)(Hglyc)$_2$(trz)$_2$(H$_2$O)] (**3**) in solid states at 90 K.

Supplementary Table 1. Crystallographic data and structural refinements for complexes [Δ/Λ-Mo$^*_2$O$_2$(μ$_2$-S)(μ$_2$-O)(*R*-Hlact*)$_2$(trz)$_2$(trz)]·½H$_2$O (**1**), [Λ/Δ-Mo$^*_2$O$_2$(μ$_2$-S)(μ$_2$-O)(*S*-Hlact*)$_2$(trz)$_2$(trz)] (**2**), and [Mo$_2$O$_2$(μ$_2$-S)(μ$_2$-O)(Hglyc)$_2$(trz)$_2$(H$_2$O)] (**3**).

Supplementary Table 2. Selected hydrogen bond distances (Å) and angles (°) in [Δ/Λ-Mo$^*_2$O$_2$(μ$_2$-S)(μ$_2$-O)(*R*-Hlact*)$_2$(trz)$_2$(trz)]·½H$_2$O (**1**).

Supplementary Table 3. Selected hydrogen bond distances (Å) and angles (°) in [Λ/Δ-Mo$^*_2$O$_2$(μ$_2$-S)(μ$_2$-O)(*S*-Hlact*)$_2$(trz)$_2$(trz)] (**2**).

Supplementary Table 4. Selected hydrogen bond distances (Å) and angles (°) in [Mo$_2$O$_2$(μ$_2$-S)(μ$_2$-O)(Hglyc)$_2$(trz)$_2$(H$_2$O)] (**3**).

Supplementary Table 5. Selected bond distances (Å) and angles (°) for [Δ/Λ-Mo$^*_2$O$_2$(μ$_2$-S)(μ$_2$-O)(*R*-Hlact*)$_2$(trz)$_2$(trz)]·½H$_2$O (**1**).

Supplementary Table 6. Selected bond distances (Å) and angles (°) for [Λ/Δ-Mo$^*_2$O$_2$(μ$_2$-S)(μ$_2$-O)(*S*-Hlact*)$_2$(trz)$_2$(trz)] (**2**).

Supplementary Table 7. Selected bond distances (Å) and angles (°) for [Mo$_2$O$_2$(μ$_2$-S)(μ$_2$-O)(Hglyc)$_2$(trz)$_2$(H$_2$O)] (**3**).

Supplementary Table 8. Detail calibrated adsorption data of O_2, N_2, H_2, CO_2 and CH_4 for [Λ/Δ-Mo$^*_2$O$_2$(μ$_2$-S)(μ$_2$-O)(*S*-Hlact*)$_2$(trz)$_2$(trz)] (**2**).

Supplementary Table 9. Detail calibrated adsorption data of O_2 and N_2 for [Δ/Λ-Mo$^*_2$O$_2$(μ$_2$-S)(μ$_2$-O)(*R*-Hlact*)$_2$(trz)$_2$(trz)]·½H$_2$O (**1**).

Supplementary Table 10. Comparisons of selected bond distances (Å) for [Δ/Λ-Mo$^*_2$O$_2$(μ$_2$-S)(μ$_2$-O)(*R*-Hlact*)$_2$(trz)$_2$(trz)]·½H$_2$O (**1**), [Λ/Δ-Mo$^*_2$O$_2$(μ$_2$-S)(μ$_2$-O)(*S*-Hlact*)$_2$(trz)$_2$(trz)] (**2**), [Mo$_2$O$_2$(μ$_2$-S)(μ$_2$-O)(Hglyc)$_2$(trz)$_2$(H$_2$O)] (**3**), (PyH)$_2$[Mo$_2$O$_4$(glyc)$_2$Py$_2$] (**4**),[1] (PyH)$_4$[Mo$_4$O$_8$Cl$_4$(glyc)$_2$]·2EtOH (**5**),[1] [Mo$_4$O$_8$(glyc)$_2$Py$_4$] (**6**),[1] K$_6$[(MoO$_2$)$_8$(glyc)$_6$(Hglyc)$_2$]·10H$_2$O (**7**),[2] *trans*-[(MoO)$_2$O(glyc)$_2$(bpy)$_2$]·3H$_2$O (**8**),[3] *trans*-[(MoO)$_2$O(*R*,*S*-lact)$_2$(bpy)$_2$]·3H$_2$O (**9**),[3] *trans*-[(MoO)$_2$O(*R*,*S*-lact)$_2$(phen)$_2$]·4H$_2$O (**10**),[3] Na$_2$[Mo$_3$(μ$_3$-S)(μ$_2$-O)$_3$(glyc)$_3$(2-mim)$_3$]·1.5H$_2$O (**11**),[4] (4-Hmim)$_6$[Mo$_3$(μ$_3$-S)(μ$_2$-O)$_3$(glyc)$_3$(4-mim)$_3$]$_2$[MoO$_2$(glyc)$_2$] (**12**),[4] Na$_3$(4-Hmim)[Mo$_3$(μ$_3$-S)(μ$_2$-O)$_3$(SO$_3$)(glyc)$_3$(4-mim)]·8H$_2$O (**13**),[4] [Mo$_3$SO$_3$(glyc)$_2$(im)$_5$]·im·H$_2$O (**14**),[5] Na$_2$[Mo$_3$SO$_3$(*R*,*S*-lact)$_3$(im)$_3$]·10H$_2$O (**15**),[5] [Mo$_3$S$_4$(PPh$_3$)$_3$(Hlact)$_2$(lact)] (**16**),[6] K$_2$[MoO$_2$(glyc)$_2$]·H$_2$O (**17**),[2] [(C6H5)4P][MoO2(glyc)(Hglyc)] (**18**),[1] [Na$_2$[MoO$_2$(*S*-lact)$_2$]]$_3$·13H$_2$O (**19**).[2]

Supplementary Table 11. The bond distances (Å) of Mo–O$_{\alpha\text{-alkoxy/hydroxy}}$, Mo–O$_{\alpha\text{-carboxy}}$ and C–O$_{\alpha\text{-alkoxy/hydroxy}}$ in FeMo-cos of nitrogenases (**20**).

Supplementary Table 12. Qualitative assignments of the most intensive bands in VCD spectra of FeMo-co **20** and K$_5$[Λ,Λ,Λ,Λ-Mo$^*_4$O$_{11}$(*R*-Hhomocit*)$_2$]Cl·5H$_2$O **21**.

Supplementary Table 13. Comparison of obtained experimental IR, VCD charactermatic signals of [Δ/Λ-Mo$^*_2$O$_2$(μ$_2$-S)(μ$_2$-O)(*R*-Hlact*)$_2$(trz)$_2$(trz)]·½H$_2$O (**1**) in the solid state and assignments of calculated VCD signals from optimized structure of **1**.

Supplementary Table 14. Comparisons of obtained experimental IR, VCD charactermatic signals of extracted FeMo-co Δ-Mo*Fe_7S_9C[*R*-(H)homocit*]$(NMF)_2$ (**20**) and assignments of calculated VCD signals from optimized structure of **20**.

Supplementary Table 15. Bond valence calculations for complexes [Δ/Λ-Mo*$_2O_2(\mu_2$-S)(μ_2-O)(*R*-Hlact*$)_2(trz)_2$(trz)]·½H_2O (**1**), [Λ/Δ-Mo*$_2O_2(\mu_2$-S)(μ_2-O)(*S*-Hlact*$)_2(trz)_2$(trz)] (**2**), and [$Mo_2O_2(\mu_2$-S)(μ_2-O)$(Hglyc)_2(trz)_2(H_2O)$] (**3**).

Part II IR spectra of 1,2,4-triazole, lactic acid, *N*-methylformamide, sodium homocitrate, $K_2[Mo^{VI}O_2(\textit{R,S}\text{-}H_2homocit)_2]\cdot 2H_2O$, and $Na_2[Mo_3SO_3(\textit{R,S}\text{-lact})_3(im)_3]\cdot 10H_2O$

Supplementary Fig. 23. FT-IR spectrum of 1,2,4-triazole originated from Spectral Database for Organic Compounds SDBS. URL for this compound: https://sdbs.db.aist.go.jp/sdbs/cgi-bin/landingpage?sdbsno=3852.

Supplementary Fig. 24. FT-IR spectrum of lactic acid originated from Spectral Database for Organic Compounds SDBS. The 1047 cm^{-1} peak is assigned to C–OH vibration. URL for this compound: http://sdbs.db.aist.go.jp/sdbs/cgi-bin/direct_frame_disp.cgi?sdbsno=12682.

Supplementary Fig. 25. FT-IR spectrum of *N*-methylformamide [C_2H_5NO] originated from Spectral Database for Organic Compounds SDBS. URL for this compound: https://sdbs.db.aist.go.jp/sdbs/cgi-bin/direct_frame_top.cgi.

Supplementary Fig. 26. FT-IR spectrum of $Na_3(Hhomocit)\cdot H_2O$[7]. The 1086 cm^{-1} peak is assigned to $C–O_H$ vibration.

Supplementary Fig. 27. FT-IR spectrum of $K_2[Mo^{VI}O_2(\textit{R,S}\text{-}H_2homocit)_2]\cdot 2H_2O$.[7] The peak at 1084 cm^{-1} is assigned to C–O vibration.

Supplementary Fig. 28. FT-IR spectrum of $Na_2[Mo_3SO_3(\textit{R,S}\text{-lact})_3(im)_3]\cdot 10H_2O$.[5] The peaks at 1049, 1060 and 1056 cm^{-1} are assigned to C–O vibration.

Supplementary Methods

Part I Syntheses, spectral characterizations and structural analyses for molybdenum *α*-hydroxycarboxylates

Physical measurements supplement. $(NH_4)_6Mo_7O_{24}\cdot4H_2O$, $Na_2MoO_4\cdot2H_2O$, *R*-lactic acid, *S*-lactic acid, glycolic acid, 1,2,4-triazole and $Na_2S_2O_4$ were purchased from Sigma. Infrared spectra were recorded as Nujol mulls between KBr plates on a Nicolet 380 FT-IR spectrometer. Solid state ^1H and ^{13}C NMR spectra were recorded on a Bruker 400 MHz Avance III SS-NMR using ADA {Tricyclo[3.3.1.1(3,7)]decane} as an external reference. Soild diffused reflectance UV-Vis spectra were recorded in solid state on a Cary 5000 spectrophotometer in the range of 200 – 800 nm at 293 K. Solid electron paramagnetic resonance (EPR) spectra were analysed by a Bruker EMX-10/12 spectrometer using crystalline samples at 298 K. TG analyses were performed on a Netzsch TG209F1 instrument in N_2 at a heating rate of 10 ^oC min^{-1}. The TG-DTA measurements were conducted on a Netzsch STA 449F5 thermal analysis system in N_2 atmosphere with a heating rate 10 ^oC min^{-1} over the 30 ~ 1000 ^oC range.

Preparations of [Λ/Δ-Mo*$_2$O$_2$(μ$_2$-S)(μ$_2$-O)(*S*-Hlact*)$_2$(trz)$_2$(trz)] (2). Synthesis of **2** was the same as that of **1**, except *S*-lactic acid (1.00 mL, 13.0 mmol) was used instead of *R*-lactic acid. Product **2** was isolated as yellow plates after one week in 43.9% yields (0.722 g) based on molybdenum. Elemental analysis (calc. for $C_{12}H_{19}Mo_2N_9O_9S$): C, 21.9; H, 2.9; N, 19.2%. Found: C, 21.8; H, 2.8; N, 19.2%. mp: 452 ℃ (decompose, molybdenum oxide). IR (KBr, cm^{-1}): ν(OH) $3448_{br,\ s}$; ν(C–H)

3127$_s$, 2941$_m$, 2856$_w$; $\nu_{as}(CO_2)$ 1638$_s$; $\nu_s(CO_2)$ 1381$_s$; ν(Mo=O) 977$_s$. UV(H_2O, nm): 322. Solid-state ^{1}H NMR (400 MHz, ADA, ppm): δ 8.56 (s, 1H), 3.95 (s, 1H), 0.52 (s, 1H). Solid-state ^{13}C NMR (400 MHz, 25 °C, ADA, ppm): δ = 179.81 (**C**O_2), 154.50 – 141.69 (trz), 70.17 (**C**OH), 19.50 (**C**H_3).

Preparation of [$Mo_2O_2(\mu_2$-S)(μ_2-O)(Hglyc)$_2$(trz)$_2$(H_2O)] (3). $(NH_4)_6Mo_7O_{24}\cdot 4H_2O$ (0.883 g, 0.72 mmol) and excess glycolic acid (1.14 g, 15.0 mmol) were dissolved in 5.0 mL water. The pH value of the solution was adjusted to 5.0 with the addition of NaOH. The mixture was heated at 60 °C for 24 hours and cooled to room temperature. Then $Na_2S_2O_4$ (0.871 g, 5.0 mmol) and 1,2,4-triazole (0.691 g, 10.0 mmol) were added in solution. The pH was justed to 3.0 with concentrated hydrochloric acid. The reactants were heated at 80 °C for 24 hours and cooled to room temperature. Product **3** was isolated as yellow plates in 66.2% yields (0.957 g) based on molybdenum. Elemental analysis (calc. for $C_8H_{14}Mo_2N_6O_{10}S$): C, 16.6; H, 2.4; N, 14.5%. Found: C, 16.5; H, 2.4; N, 14.6%. mp: 467 ℃ (decompose, molybdenum oxide). IR (KBr, cm^{-1}): ν(OH) 3460$_s$; ν(C–H) 3141$_s$, 2951$_m$, 2563$_w$; $\nu_{as}(CO_2)$ 1655$_{vs}$; $\nu_s(CO_2)$ 1359$_s$; ν(Mo=O) 967$_s$. UV(H_2O, nm): 250. Solid-state ^{1}H NMR (400 MHz, ADA, ppm): δ 8.41 (s, 1H), 4.92 (s, 2H). Solid-state ^{13}C NMR (400 MHz, 25 °C, ADA, ppm): δ = 177.87 (**C**O_2), 151.65 – 142.26 (trz), 62.86 (**C**OH).

Preparation of {Na_2[Δ-Mo$^*O_2$(*R*-lact*)$_2$]}$_3\cdot 13H_2O$.[2] Sodium molybdate dihydrate (1.21 g, 5.0 mmol) and *R*-lactic acid (1.00 ml, 13.0 mmol) were dissolved in 5.0 ml water. pH value was adjusted to 4.0 with dilute NaOH. The solution was heated at 60 °C for 5 h. The compound was separated as colorless crystals after three days and they

were collected and washed with ethanol. yield: 67.9%. Similarly, $\{Na_2[\Lambda\text{-}Mo^*O_2(S\text{-lact}^*)_2]\}_3\cdot 13H_2O$ was obtained.[2]

Preparation of $K_5[\Lambda,\Lambda,\Lambda,\Lambda\text{-}Mo^*_4O_{11}(R\text{-Hhomocit}^*)_2]Cl\cdot 5H_2O$ (21).[8] *R*-homocitric *γ*-lactone acid (14.0 mg, 0.07 mmol), prepared by the reported method[9] was dissolved in a minimal amount of water. The pH was adjusted by KOH to 11 to generate the acyclic homocitrate as monitored with HPLC. After hydrolysis, potassium molybdate (95 mg, 0.40 mmol) was added in small portions over 10 minutes. Upon cooling in an ice bath for 15 min, the pH of the solution was adjusted to 2.0 with hydrochloric acid to induce complex formation. The salts of chloride were filtrated with precipitation. Complex **21** was formed after sitting at room temperature for four days. Yield: 0.30 mg.

Gas Adsorption Measurements. The gas adsorption capacities of **1** ~ **2** were evaluated with a magnetic suspension gravimetric sorption analyzer (ISOSORP-HTGRA) at 298 K at different pressures. Gases adsorption isotherms for pressures up to 30 bar at 298 K was obtained on a magnetic suspension balance (MSB) (RUBOTHERM ISOSORP-HTGRA) operated by MessPro software. In a typical experiment, like the sample **2**, about 112.4 mg, was placed in the sample bucket and loaded in the absorption chamber. The sample was then evacuated at 423 K to remove any traces of water, solvents or dissolved gases until the weight remains constant. The chamber was then pressurized with dry O_2 at 298 K and the change in weight was monitored by MSB. Each step with different pressure took around 40 min until the adsorption stabilized.

X-Ray Crystallography. The crystal data for **1** ~ **3** were collected on an Oxford Gemini CCD diffractometer, with graphite monochromatic Mo-Kα radiation (λ = 0.71073 Å) at 173 K. Muti-scan absorption corrections were applied. The structures were solved by ShelXT and refined by full-matrix least-squares on F_2 using the $OLEX_2$ crystallographic software package.[10-12] All non-hydrogen atoms were refined anisotropically, while hydrogen atoms were generated geometrically or located from differential Fourier maps and refined isotropically. Crystallographic data and structural refinements for complexes **1** ~ **3** are listed in Supplementary Table 1. CCDC deposition numbers are 1993896 − 1993898.

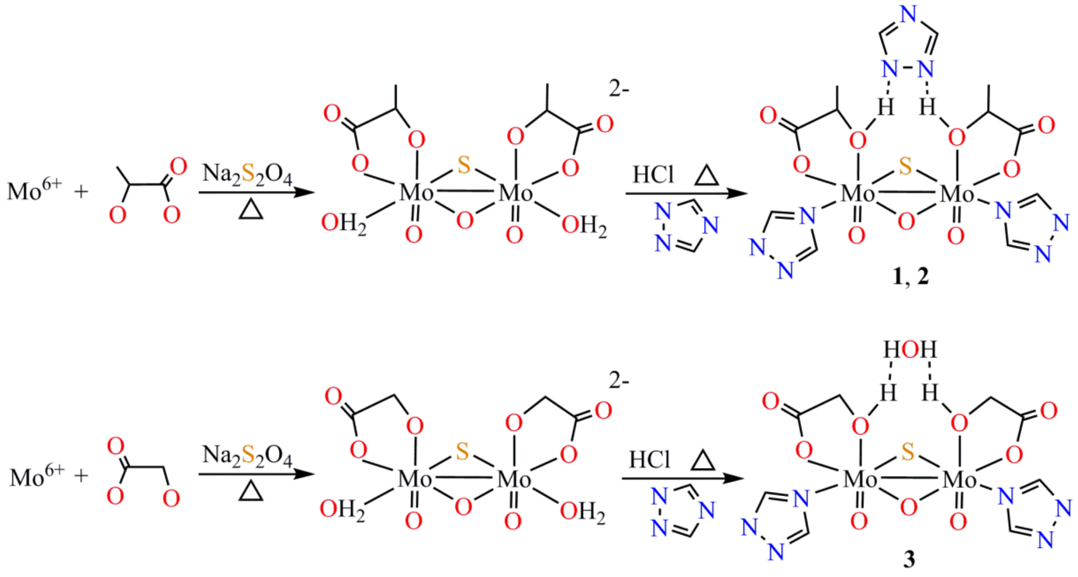

Supplementary Fig. 1. Syntheses and possible transformations of dinuclear oxidomolybdenum(V) triazole lactates **1**, **2** and glycolates **3** respectively.

Syntheses. Preparations of **1** ~ **3** mainly depend on the requisite proportions of molybdate and ligands, countercations, pH and the reaction temperature. Compared with the deprotonated molybdenum *α*-hydroxypolycarboxylates reported,[2,8,13-22] protonated molybdenum lactates and glycolates in **1** ~ **3** may be attributed to the strong acidity of the solution and multiple *N*-coordination sites with 1,2,4-triazole to stabilize the structures. The favorable pH value was in the range of 2.0 ~ 3.0, increase of pH will lead to failure of the reactions.

Possible syntheses and transformations of oxidomolybdenum(V) triazole lactates and glycolates **1** ~ **3** are outlined in Supplementary Fig. 1. Firstly, dinuclear molybdenum(V) lactate or glycolate $[Mo_2O_2(\mu_2\text{-}O)(\mu_2\text{-}S)L_2(H_2O)_2]^{2-}$ (L = lactate/glycolate) formed in reduced solutions with $Na_2S_2O_4$, which are similar to molybdenum oxalate $[Mo_2O_4(ox)_2(H_2O)_2]^{2-}$ reported.[23] Further substitutions of coordinated water molecules with 1,2,4-triazoles and acidification of lactate or glycolate result in the formations of the final neutral products **1** ~ **3** respectively.

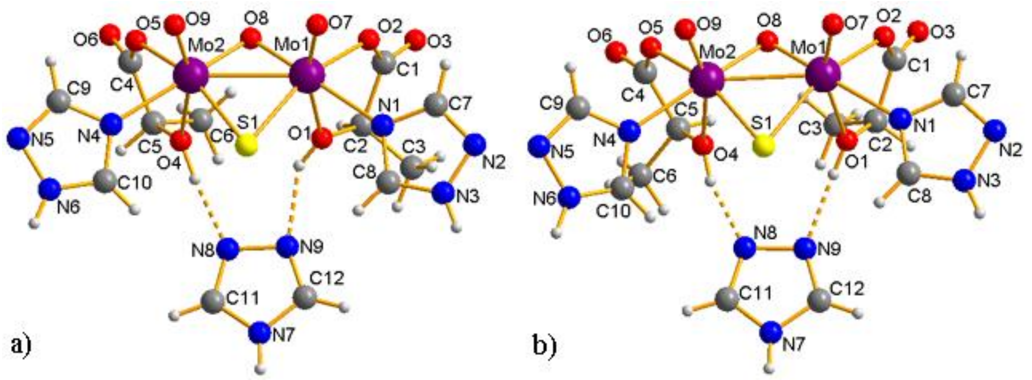

Supplementary Fig. 2. Molecular structures of [Δ/Λ-Mo*$_2$O$_2$(μ$_2$-S)(μ$_2$-O)(*R*-Hlact*)$_2$(trz)$_2$(trz)]·½H$_2$O (**1**, a) and its enantiomer [Λ/Δ-Mo*$_2$O$_2$(μ$_2$-S)(μ$_2$-O)(*S*-Hlact*)$_2$(trz)$_2$(trz)] (**2**, b), free water molecule in **1** was omitted for clarity.

Structural analyses of 1 ~ 2. As shown in Supplementary Fig. 2, X-ray crystal structures of [Δ/Λ-Mo*$_2$O$_2$(μ$_2$-S)(μ$_2$-O)(*R*-Hlact*)$_2$(trz)$_2$(trz)]·½H$_2$O (**1**) and [Λ/Δ-Mo*$_2$O$_2$(μ$_2$-S)(μ$_2$-O)(*S*-Hlact*)$_2$(trz)$_2$(trz)] (**2**) show a pair of enantiomers with dinuclear molybdenum(V) 1,2,4-triazole lactates. The main structure is the same except for chiral configurations of molybdenum(V) lactates and the number of free water molecules. For **1**, its central core consists of two hexa-coordinated molybdenum atoms in *quasi*-octahedral geometries. The molybdenum(V) atoms are bridged by sulfur and oxygen, and the other corners were completed by *R*-lactates and 1,2,4-triazoles. The lactates in **1** and **2** chelate to molybdenum atoms with *α*-hydroxy and *α*-carboxy groups respectively. Triazole coordinates to molybdenum atom with nitrogen as monodentate ligand. Free triazole acts as a bridge to connect the dinuclear unit through strong hydrogen bonds with hydroxy groups of lactates [O(1)$_{\alpha\text{-hydroxy}}$···N(9) 2.617(1) Å, O(4)$_{\alpha\text{-hydroxy}}$···N(8) 2.718(1) Å in **1**, O(1)$_{\alpha\text{-hydroxy}}$···N(9) 2.717(8) Å, O(4)$_{\alpha\text{-hydroxy}}$···N(8) 2.609(7) Å in **2**]. This is important to stabilize the protonated structures in **1** and **2**.

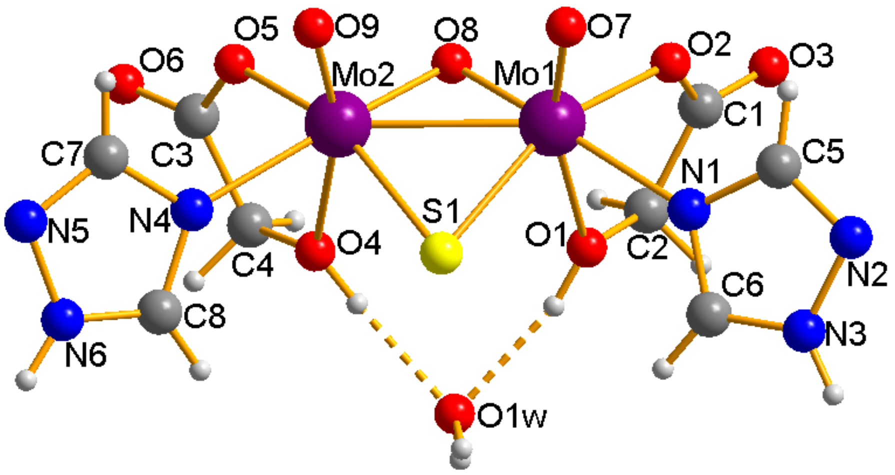

Supplementary Fig. 3. Molecular structure of $[Mo_2O_2(\mu_2\text{-}S)(\mu_2\text{-}O)(Hglyc)_2(trz)_2(H_2O)]$ (**3**).

Structural analysis of 3. $[Mo_2O_2(\mu_2\text{-}S)(\mu_2\text{-}O)(Hglyc)_2(trz)_2(H_2O)]$ (**3**) is a dinuclear mixed ligand molybdenum(V) glycolate as shown in Supplementary Fig. 3. Its main structure is similar to **1**. In **3**, each molybdenum atom is surrounded octahedrally by one μ_2-S and one μ_2-O, one N atom of triazole, two O atoms (α-hydroxy and α-carboxy) of glycolate. Free water molecule becomes bridge in the molecule *via* strong hydrogen bonds [$O(1)_{\alpha\text{-hydroxy}}\cdot\cdot O(1w)$ 2.675(9) Å, $O(4)_{\alpha\text{-hydroxy}}\cdot\cdot O(1w)$ 2.675(9) Å]. Unlike **1** or **2**, the protonated structure is stabilized by free water molecule O1w instead of 1,2,4-triazole. The other intermolecular hydrogen bonds are listed in Supplementary Table 4 like O(1w)··O(8)a 2.670(9) Å *a* (x, $2 - y$, $½ + z$); O(1w)··O(7) 3.051(9) Å.

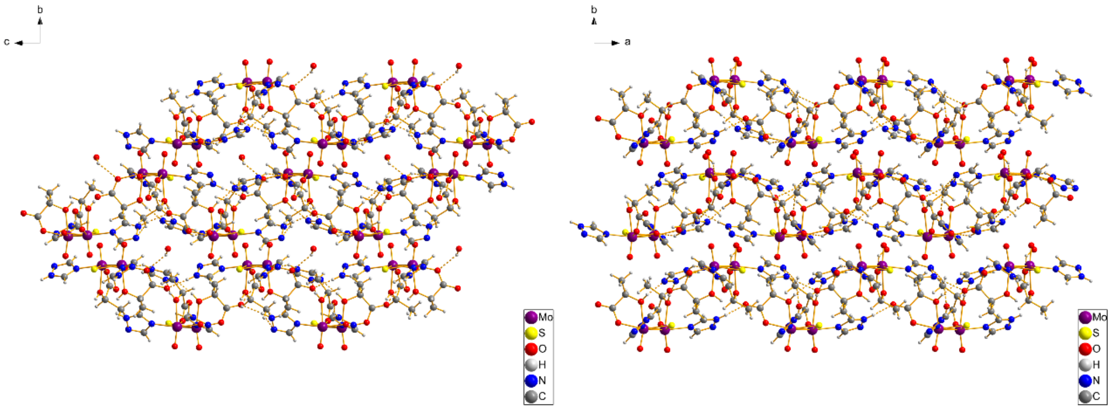

Supplementary Fig. 4. 2D layered diagrams in $[\Delta/\Lambda\text{-}\mathrm{Mo}^{*}{}_{2}\mathrm{O}_{2}(\mu_{2}\text{-}\mathrm{S})(\mu_{2}\text{-}\mathrm{O})(R\text{-}\mathrm{Hlact}^{*})_{2}(\mathrm{trz})_{2}(\mathrm{trz})]\cdot\frac{1}{2}\mathrm{H_2O}$ (**1**).

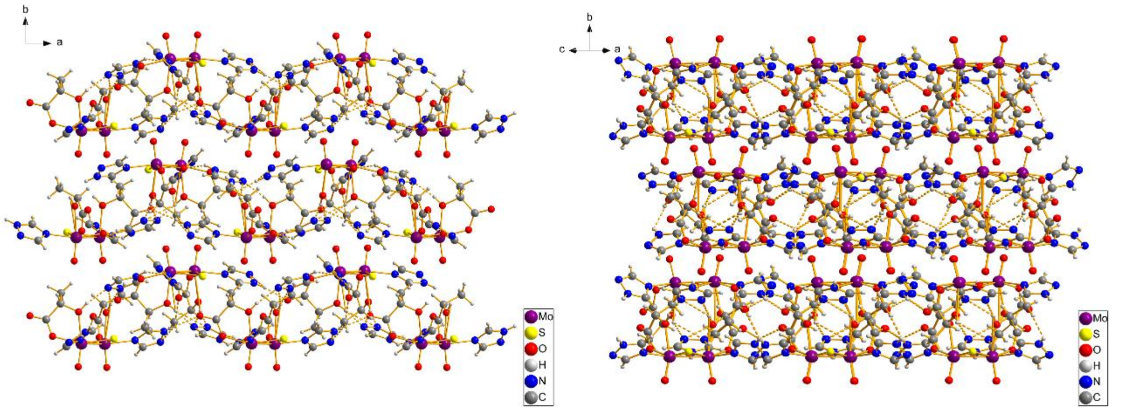

Supplementary Fig. 5. 2D layered diagrams in [Λ/Δ-Mo*$_2$O$_2$(μ$_2$-S)(μ$_2$-O)(*S*-Hlact*)$_2$(trz)$_2$(trz)] (**2**).

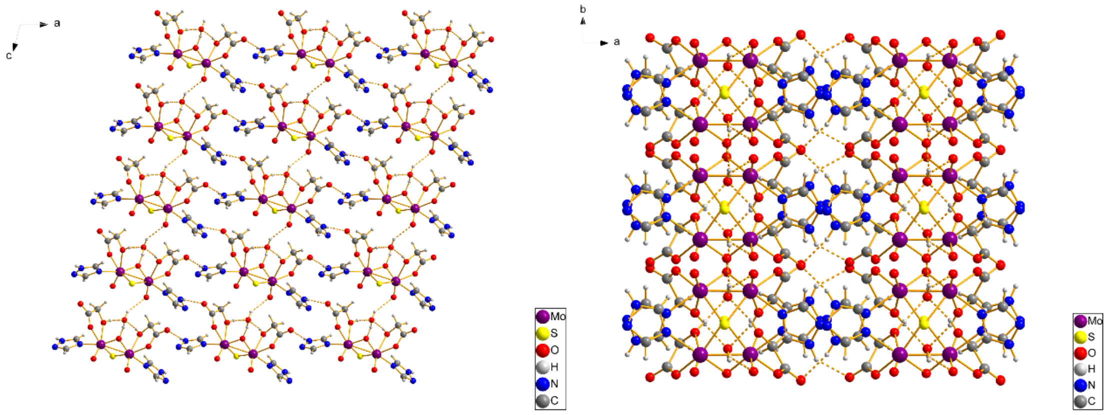

Supplementary Fig. 6. 2D layered diagrams in $[Mo_2O_2(\mu_2\text{-}S)(\mu_2\text{-}O)(Hglyc)_2(trz)_2(H_2O)]$ (**3**).

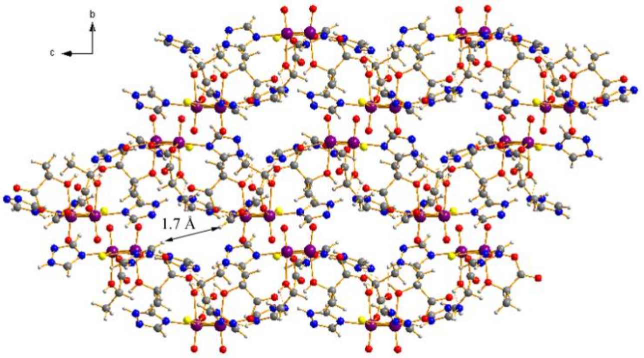

Supplementary Fig. 7. The channels existed in [Λ/Δ-$Mo^*_2O_2(\mu_2$-S)(μ_2-O)(*S*-$Hlact^*)_2(trz)_2$(trz)] (**2**) viewed along *a* axis.

Channels description in 2. The minimum inner diameter of the channels existed in **2** is 1.7 Å. The holes exhibit regular oval shape with varying lengths (approximately 5.5 Å long and 2.6 Å wide). The channels are formed by the accumulation of skeletons, and are created by multiple hydrogen bonds between 1,2,4-triazoles and lactates. The smallest constituent unit of the channel includes 12 Mo^{5+}.

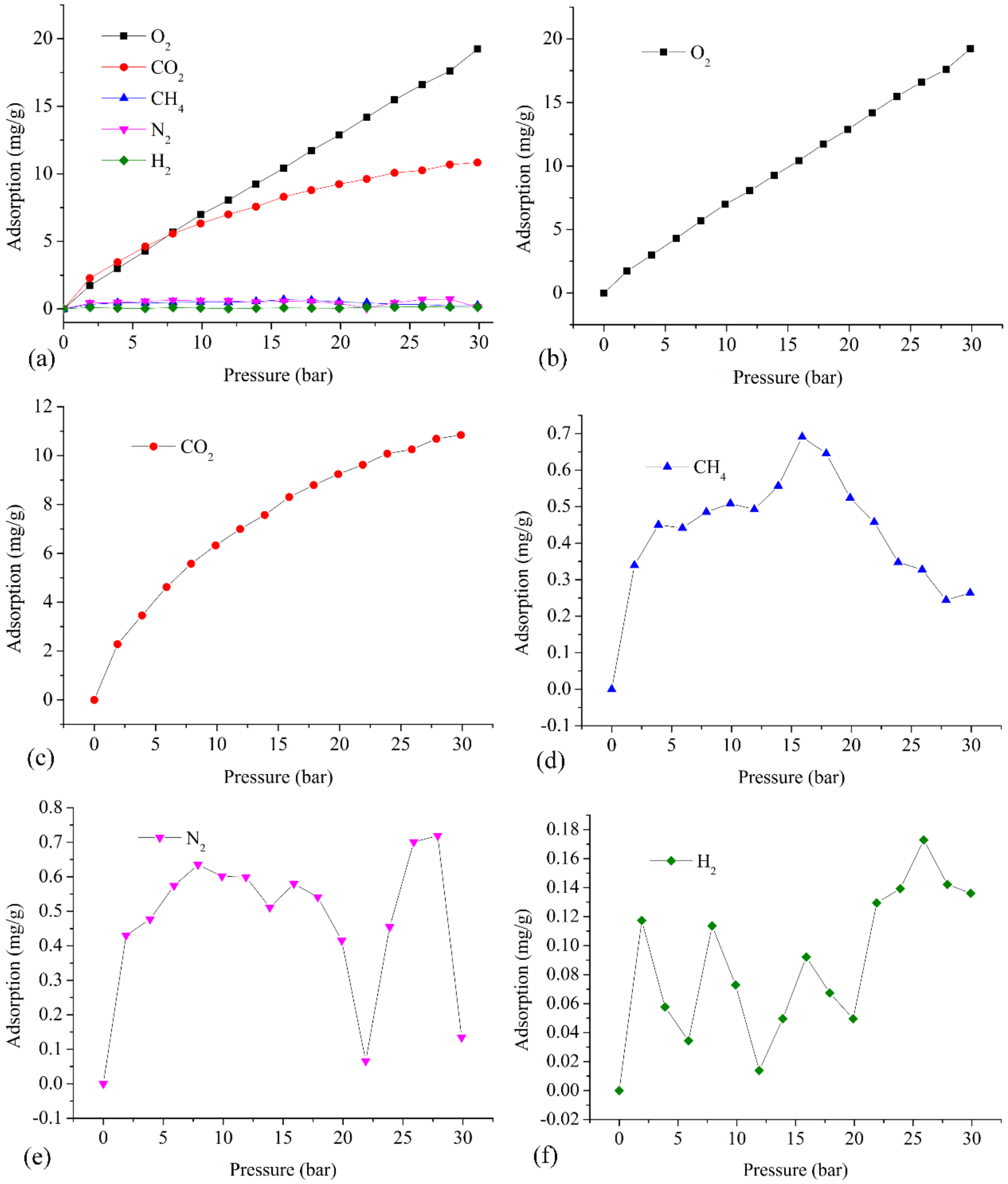

Supplementary Fig. 8. (a) Adsorption isotherms of **2** for the gases of O_2, CO_2, CH_4, N_2 and H_2 at 298 K at different pressures. Independent O_2 (b), CO_2 (c), CH_4 (d), N_2 (e) and H_2 (f) adsorption isotherms of [Λ/Δ-$Mo^*{}_2O_2(\mu_2\text{-}S)(\mu_2\text{-}O)$(*S*-Hlact*)$_2(trz)_2$(trz)] (**2**) at 298 K in different pressures.

Adsorption behavior of 2. There is a small channel (diameter 1.7 Å) existed in the 3D structure of **2** viewed along *a* axis as shown in Supplementary Fig. 7. **2** have been synthesized largely to use for the gas adsorptions of O_2, CO_2, CH_4, N_2, and H_2 at 298 K respectively. The results show that the biogases O_2 and CO_2 can be selectively

adsorbed which can be seen in Supplementary Fig. 8a. As the pressure increases, the amount of O_2 adsorbed increases rapidly with 1.72, 5.68, 10.42 and 19.23 mg/g at 1.9, 7.9, 15.9 and 29.9 bar respectively, which have a tendency to attract more as the pressure increases which are shown in Supplementary Fig. 8b. As for CO_2, the plot in Supplementary Fig. 8c shows an andante increase of CO_2 adsorption as the pressure increases, almost reaching a maximum adsorption value of 10.84 mg/g at 29.9 bar, which is the well-known Langmuir adsorption isotherm (Type I).[24] The other three gases have only little or even no adsorption, which can be seen in Supplementary Figs. 8d ~ 8f. It is noted that the higher selectivities could be obtained at higher pressures, thus **2** could be a promising sorbent for high-pressure air separation.[25] Detail calibrated adsorption data were given in Supplementary Table 8.

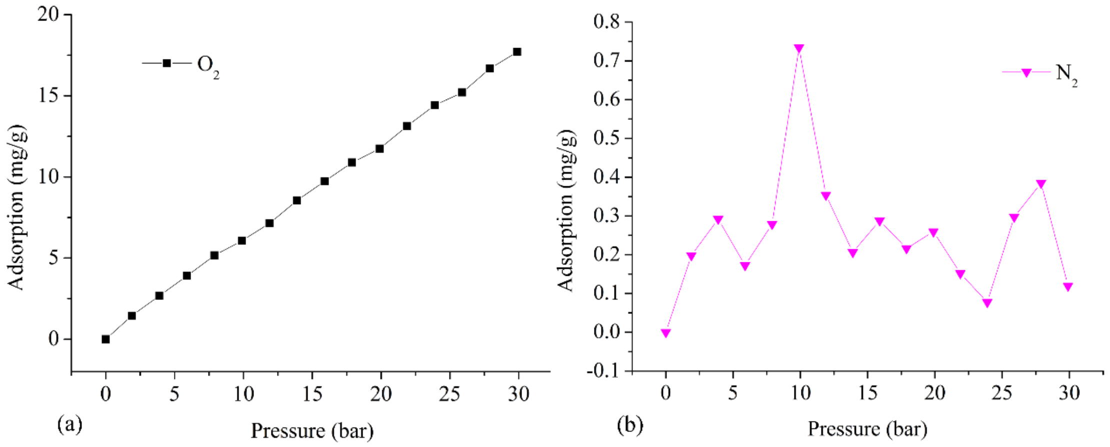

Supplementary Fig. 9. Independent O_2 (a) and N_2 (b) adsorption isotherms of [Δ/Λ-$Mo^*_2O_2(\mu_2$-S)(μ_2-O)(*R*-$Hlact^*)_2(trz)_2(trz)]\cdot½H_2O$ (**1**) at 298 K in different pressures.

Adsorption behavior of 1. Similarly, there is an analogous channel existed in the 3D structure of **1** viewed along *a* axis as shown in Supplementary Fig. 4, while a free water molecule existed in the center of the channel through hydrogen bonds. By comparing the O_2 and N_2 adsorption behavior in **1**, **2** in Supplementary Figs. 8 ~ 9, we can find the water molecules in the pores will affect the adsorption performance. Detail calibrated adsorption data for **1** were given in Supplementary Table 9.

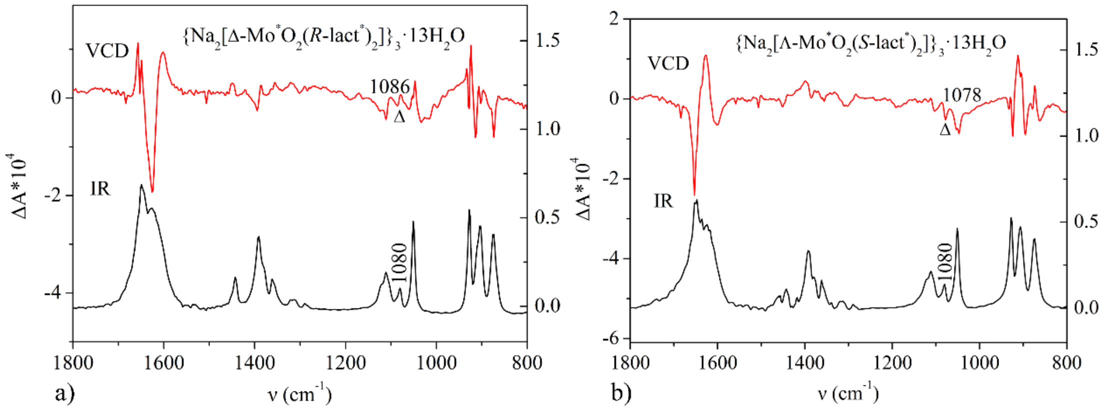

Supplementary Fig. 10. VCD and IR spectra of $\{Na_2[\Delta\text{-}Mo^*O_2(R\text{-lact}^*)_2]\}_3 \cdot 13H_2O$ (a) and $\{Na_2[\Lambda\text{-}Mo^*O_2(S\text{-lact}^*)_2]\}_3 \cdot 13H_2O$ (b) in the regions of 1800 − 800 cm^{-1}.

Analyses of VCD spectra. The obtained VCD signals of a pair of enantiomers of deprotonated $\{Na_2[\Delta\text{-}Mo^*O_2(R\text{-lact}^*)_2]\}_3 \cdot 13H_2O$ (a), $\{Na_2[\Lambda\text{-}Mo^*O_2(S\text{-lact}^*)_2]\}_3 \cdot 13H_2O$ (b) were mirror images in the regions of 1800 − 800 cm^{-1} as shown in Supplementary Fig. 10. The experimentally obtained VCD signals mostly agreed with the acquired IR signals simultaneously. VCD peaks at 1086 cm^{-1} in $\{Na_2[\Delta\text{-}Mo^*O_2(R\text{-lact}^*)_2]\}_3 \cdot 13H_2O$ and 1078 cm^{-1} in $\{Na_2[\Lambda\text{-}Mo^*O_2(S\text{-lact}^*)_2]\}_3 \cdot 13H_2O$ are assigned to the deprotonated C−O stretching vibrations based on the reported IR spectra of some alcohols and α-hydroxycarboxylic acids.[26]

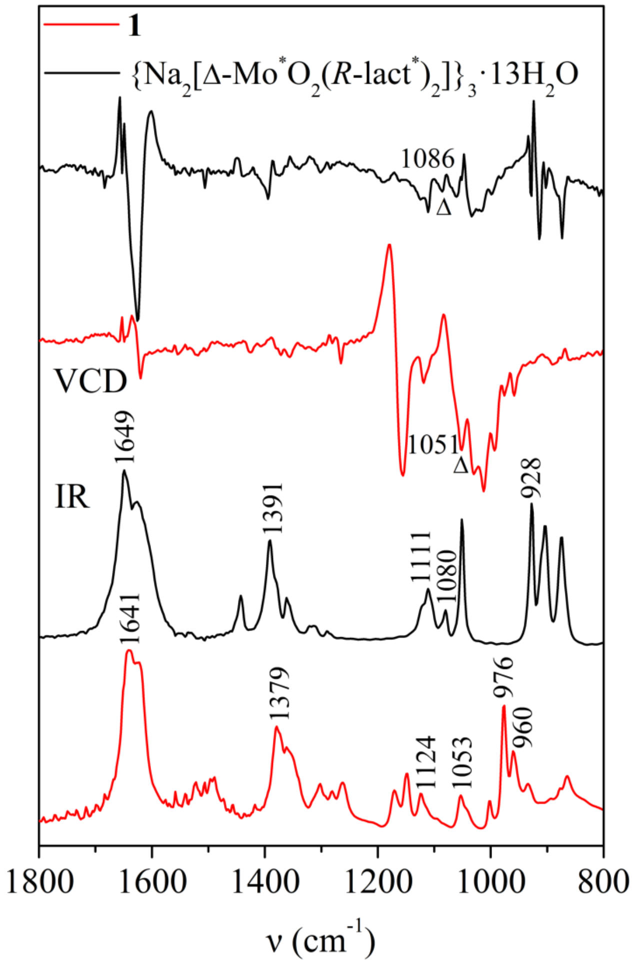

Supplementary Fig. 11. VCD and IR spectra of [Δ/Λ-Mo$^*{}_2$O$_2$(μ$_2$-S)(μ$_2$-O)(R-Hlact*)$_2$(trz)$_2$(trz)]·½H$_2$O (**1**) and {Na$_2$[Δ-Mo*O$_2$(R-lact*)$_2$]}$_3$·13H$_2$O using KBr pellet in the regions of 1800 – 800 cm^{-1}.

Analyses of VCD and IR spectra. By contrast VCD and IR spectra of **1** and {Na$_2$[Δ-Mo*O$_2$(R-lact*)$_2$]}$_3$·13H$_2$O, we can see ν_{as}(COO) peaks in them both appeared around 1650 cm^{-1}, ν_s(COO) and ν(Mo=O) signals have about 10 wave number differences or more. The peaks around 1120 cm^{-1} are identified as ν(C–N). There will be some differences in the characteristic peaks of different compounds.

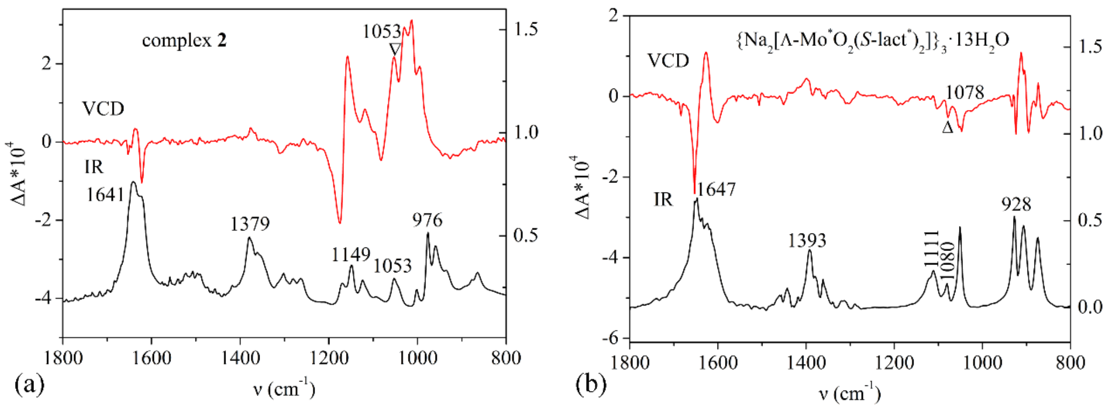

Supplementary Fig. 12. Comparisons of VCD and IR spectra of [Λ/Δ-$Mo^*{}_2O_2(\mu_2$-S)(μ_2-O)(*S*-$Hlact^*)_2(trz)_2$(trz)] (**2**, a) and {Na_2[Λ-Mo^*O_2(*S*-$lact^*)_2$]}$_3$·$13H_2O$ (b) using KBr pellet in the regions of 1800 – 800 cm^{-1}.

Analyses of VCD and IR spectra. By comparison Supplementary Fig. 12a with 12b, we can come to the same conclusion as the Supplementary Fig. 11 for similar ν_{as}(COO), ν(C–N) peaks, unlike ν_s(COO) and ν(Mo=O) signals in **2** and {Na_2[Λ-Mo^*O_2(*S*-$lact^*)_2$]}$_3$·$13H_2O$.

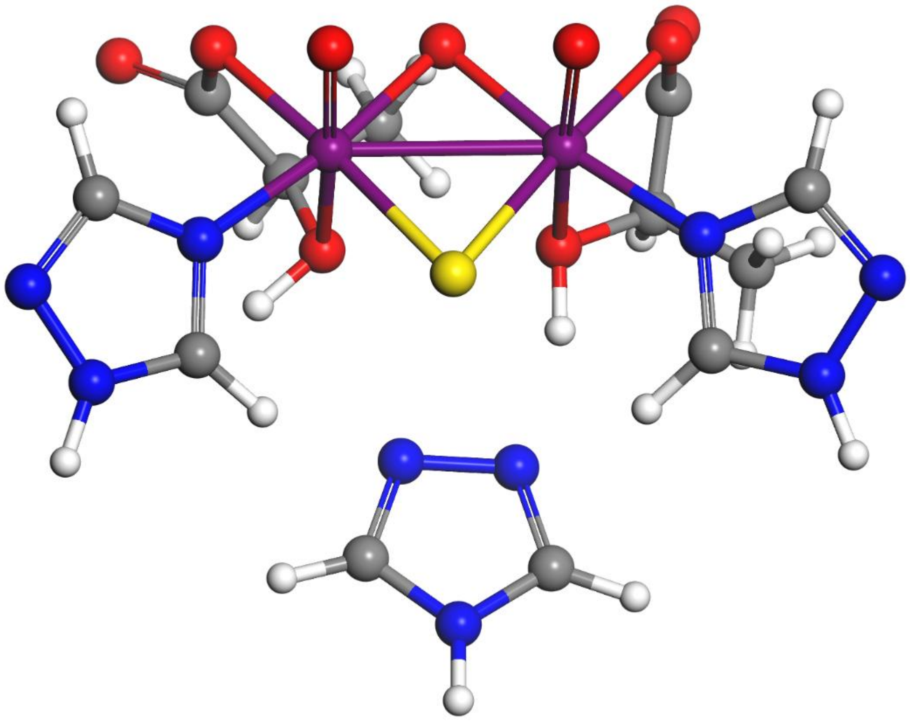

Supplementary Fig. 13. Optimized molecular structure of [Δ/Λ-$Mo^{*}{}_2O_2(\mu_2$-S)(μ_2-O)(*R*-$Hlact^{*})_2$(trz)$_2$(trz)]·½H_2O (**1**). Color: molybdenum, violet; oxygen, red; nitrogen, blue; carbon, gray; sulfur, yellow; hydrogen, white.

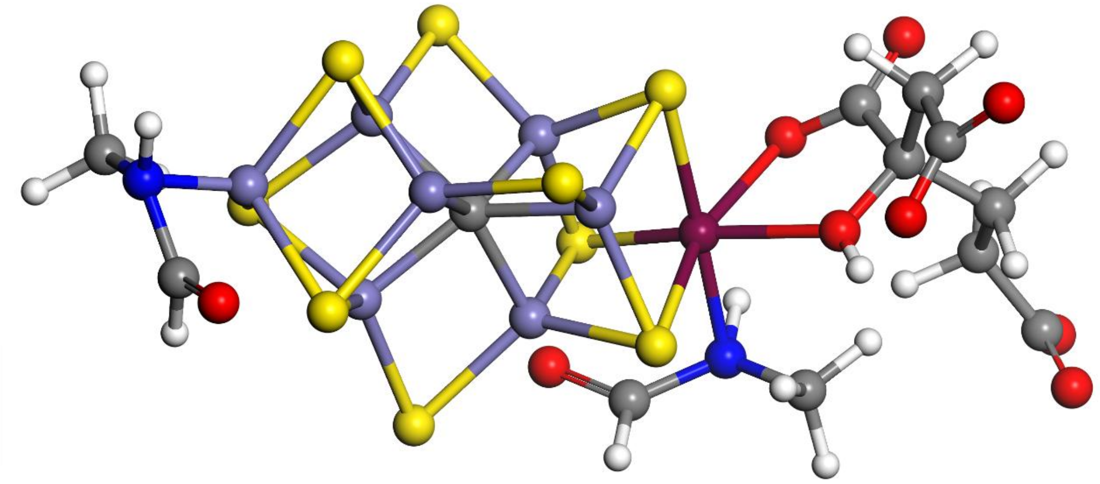

Supplementary Fig. 14. Optimized molecular structure of Δ-Mo*Fe_7S_9C[*R*-(H)homocit*]$(NMF)_2$ (**20**). Color: molybdenum, violet; oxygen, red; nitrogen, blue; carbon, gray; sulfur, yellow; hydrogen, white; iron, slate.

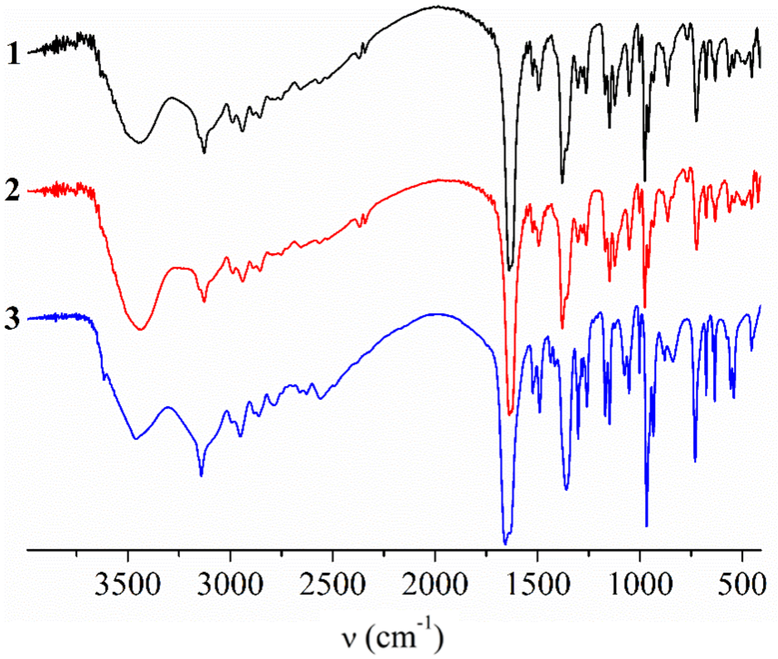

Supplementary Fig. 15. IR spectra of [Δ/Λ-Mo*$_2$O$_2$(μ$_2$-S)(μ$_2$-O)(*R*-Hlact*)$_2$(trz)$_2$(trz)]·½H_2O (**1**), [Λ/Δ-Mo*$_2$O$_2$(μ$_2$-S)(μ$_2$-O)(*S*-Hlact*)$_2$(trz)$_2$(trz)] (**2**), and [Mo$_2$O$_2$(μ$_2$-S)(μ$_2$-O)(Hglyc)$_2$(trz)$_2$(H$_2$O)] (**3**), in the region of 4000 ~ 450 cm^{-1}.

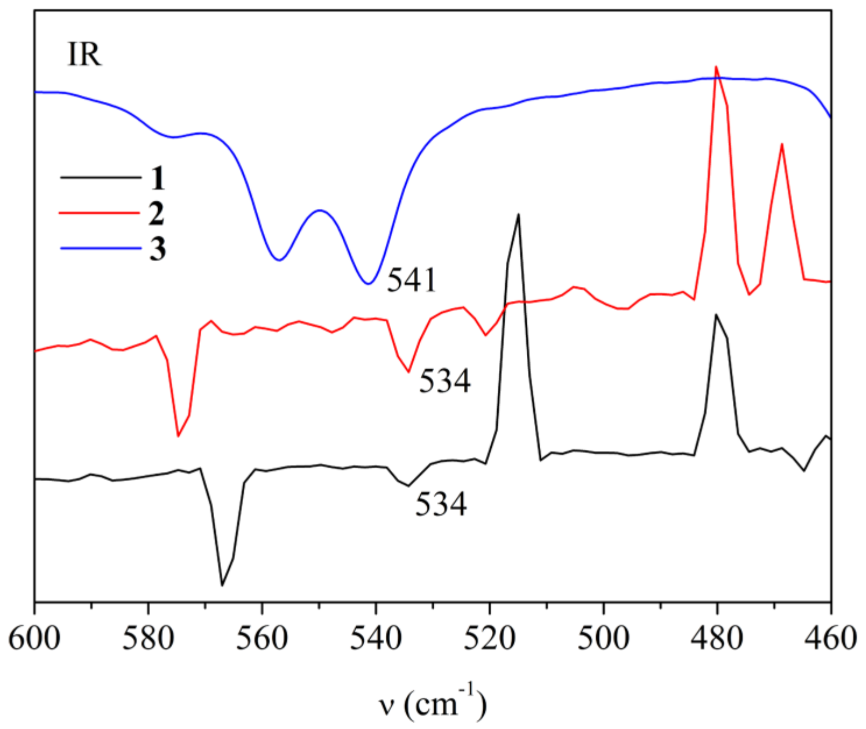

Supplementary Fig. 16. IR spectra of [Δ/Λ-Mo*$_2$O$_2$(μ$_2$-S)(μ$_2$-O)(*R*-Hlact*)$_2$(trz)$_2$(trz)]·½H_2O (**1**), [Λ/Δ-Mo*$_2$O$_2$(μ$_2$-S)(μ$_2$-O)(*S*-Hlact*)$_2$(trz)$_2$(trz)] (**2**) and [Mo$_2$O$_2$(μ$_2$-S)(μ$_2$-O)(Hglyc)$_2$(trz)$_2$(H_2O)] (**3**), in the region of 600 ~ 460 cm^{-1}.

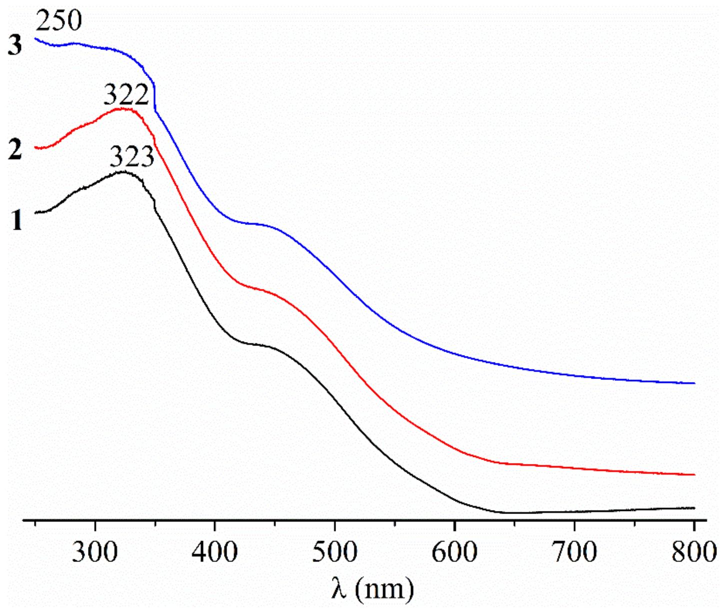

Supplementary Fig. 17. Soild diffused reflectance UV-Vis spectra of solids [Δ/Λ-$Mo^{*}_2O_2(\mu_2$-S)(μ_2-O)(*R*-$Hlact^{*})_2(trz)_2(trz)]\cdot½H_2O$ (**1**), [Λ/Δ-$Mo^{*}_2O_2(\mu_2$-S)(μ_2-O)(*S*-$Hlact^{*})_2(trz)_2(trz)]$ (**2**), and [$Mo_2O_2(\mu_2$-S)(μ_2-O)$(Hglyc)_2(trz)_2(H_2O)$] (**3**).

IR and UV spectroscopy analyses. 1 ~ **3** have been characterized by IR spectroscopies. Based on the spectra shown in Supplementary Fig. 15, vibrations of asymmetric and symmetric coordinated carboxy groups appear at 1655 ~ 1638 cm^{-1} and 1379 ~ 1359 cm^{-1} respectively. The frequency differences $\Delta[\nu_{as}(COO^{-})–\nu_{s}(COO^{-})]$[27] are greater than 200 cm^{-1}, which are consistent with the monodentate coordinated carboxy group, and in agreement with **1** ~ **3** observed from X-ray structural analyses. The vibrations of Mo=O appear at 977 ~ 967 cm^{-1}. At lower frequencies, the IR bands 534 cm^{-1} in **1**, **2** and 541 cm^{-1} in **3** are assigned to Mo–O vibrations which can be seen in Supplementary Fig. 16.[20] In UV-Vis spectra, strong absorption peaks are attribute to ligand-based π-π* transitions and charge-transfer transition of Mo–O (250 ~ 323 nm in **1** ~ **3**) as shown in Supplementary Fig. 17.

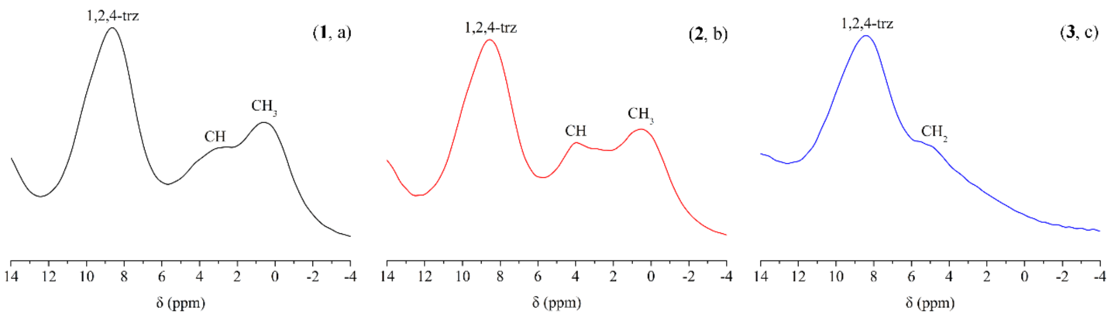

Supplementary Fig. 18. Solid-state ^{1}H NMR spectra of [Δ/Λ-Mo$^*{}_2$O$_2$(μ$_2$-S)(μ$_2$-O)(*R*-Hlact*)$_2$(trz)$_2$(trz)]·½H$_2$O (**1**, a), [Λ/Δ-Mo$^*{}_2$O$_2$(μ$_2$-S)(μ$_2$-O)(*S*-Hlact*)$_2$(trz)$_2$(trz)] (**2**, b) and [Mo$_2$O$_2$(μ$_2$-S)(μ$_2$-O)(Hglyc)$_2$(trz)$_2$(H$_2$O)] (**3**, c).

Solid ^{1}H NMR spectroscopy analyses. Solid ^{1}H NMR spectra can provide valuable information for the coordination environments and chemical behaviors of new compounds **1** ~ **3** as shown in Supplementary Fig. 18. But the resolution of the solid ^{1}H spectrum is poor, it is not easy to fit or calibrate and the characteristic peak curve is not sharp compared with ^{13}C NMR spectra in Supplementary Fig. 19. The identification of the characteristic peaks in **1** ~ **3** have been listed in the synthetic section.

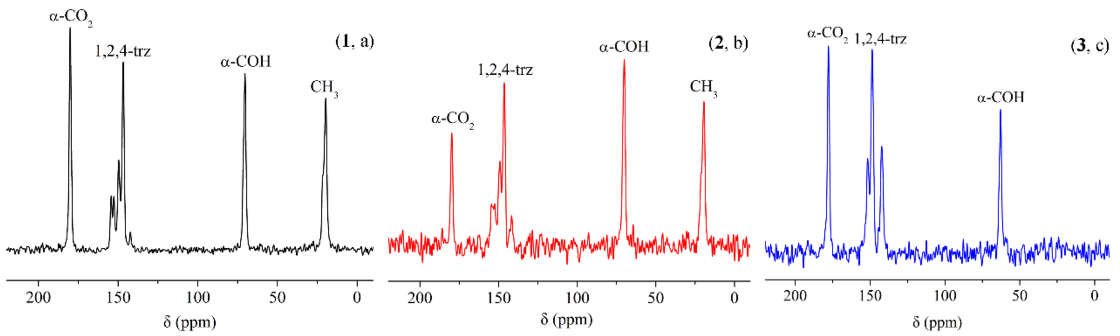

Supplementary Fig. 19. Solid-state ^{13}C NMR spectra of [Δ/Λ-Mo$^{*}_{2}$O$_2$(μ$_2$-S)(μ$_2$-O)(*R*-Hlact*)$_2$(trz)$_2$(trz)]·½H$_2$O (**1**, a), [Λ/Δ-Mo$^{*}_{2}$O$_2$(μ$_2$-S)(μ$_2$-O)(*S*-Hlact*)$_2$(trz)$_2$(trz)] (**2**, b) and [Mo$_2$O$_2$(μ$_2$-S)(μ$_2$-O)(Hglyc)$_2$(trz)$_2$(H$_2$O)] (**3**, c).

Solid ^{13}C NMR spectroscopy analyses. Solid ^{13}C NMR spectra provide valuable information for the coordination environments and chemical behaviors of **1** ~ **3** as shown in Supplementary Fig. 19. The coordinated *α*-CO$_2$ groups (180.11 ppm in **1**, 179.81 ppm in **2** and 177.87 ppm in **3**) and the coordinated *α*-hydroxy groups (70.40 ppm in **1**, 70.17 ppm in **2** and 62.86 ppm in **3**) show obvious downfield shifts compared with free lactic acid (*α*-CO$_2$, 176.18 ppm; *α*-CO, 65.68 ppm) and glycolic acid (*α*-CO$_2$, 177.04 ppm; *α*-CO, 60.16 ppm) respectively. Due to the asymmetric coordination environments, 1,2,4-triazoles in **1** ~ **3** appear as multiple carbon signals (154.38 ~ 142.28 ppm in **1**, 154.50 ~ 141.69 ppm in **2** and 151.65 ~ 142.26 ppm in **3**). The coordinated triazoles also show obvious downfield shifts compared with free 1,2,4-triazole (146.91 ppm). However, attempt for the solid-state NMR spectrum of the extracted FeMo-co *via* long time superposition is unsuccessful due to strong paramagnetic effect.

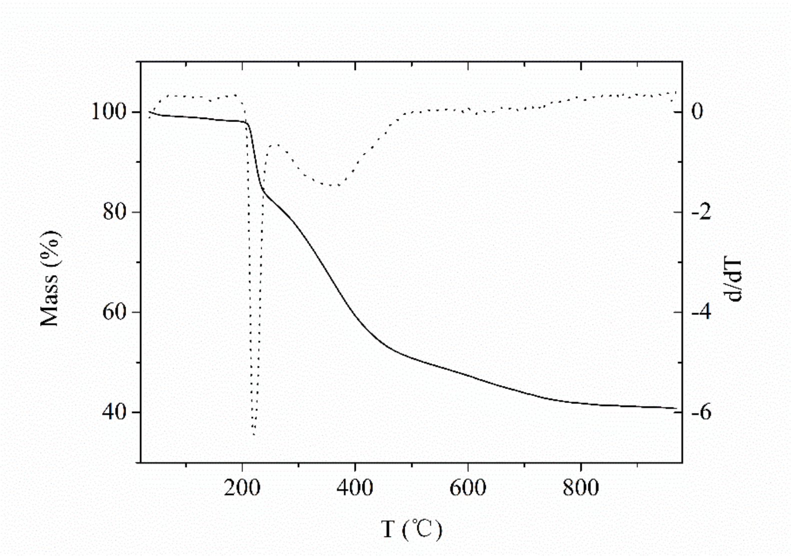

Supplementary Fig. 20a. TG–DTG curves of [Δ/Λ-Mo$^{*}{}_2$O$_2$(μ$_2$-S)(μ$_2$-O)(*R*-Hlact*)$_2$(trz)$_2$(trz)]·½H$_2$O (**1**).

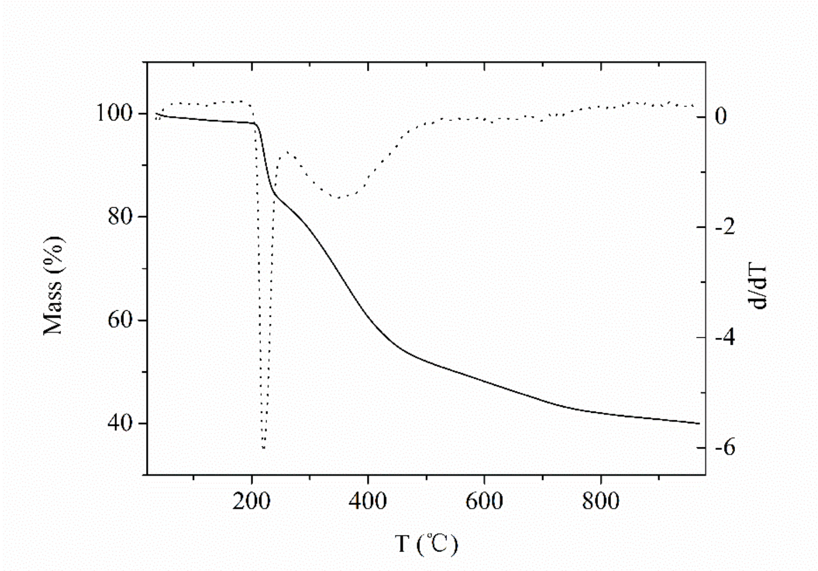

Supplementary Fig. 20b. TG–DTG curves of [Λ/Δ-$Mo^{*}_2O_2(\mu_2\text{-}S)(\mu_2\text{-}O)$(*S*-$Hlact^{*})_2(trz)_2(trz)$] (**2**).

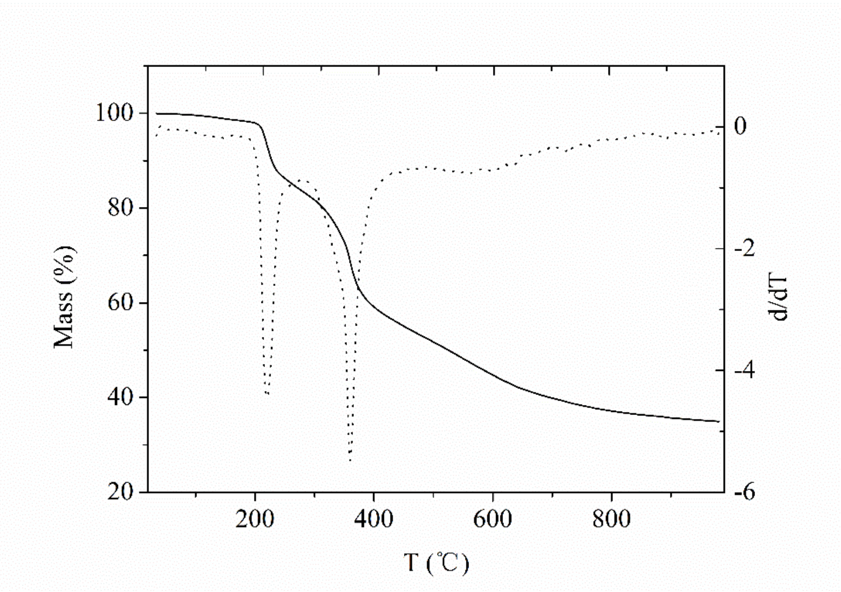

Supplementary Fig. 20c. TG–DTG curves of $[Mo_2O_2(\mu_2\text{-}S)(\mu_2\text{-}O)(Hglyc)_2(trz)_2(H_2O)]$ (**3**).

TG analyses. Thermal stabilities and decomposition patterns of **1** ~ **3** were investigated by thermogravimetric analyses. TG-DTG curves are shown in Supplementary Figs. 20a ~ 20c. Decompositions of anhydrous **1** ~ **3** occur at 220.3 ^{o}C, 220.3 ^{o}C and 220.1 ^{o}C respectively, which correspond to the weight losses of 1,2,4-triazoles. The mass % losses from 265 to 490 ℃ (31.80 % for **1**), 263 to 489 ℃ (26.71 % for **2**) and 276 to 451 ℃ (48.95 % for **3**) correspond to the mass % of C and H associated with the functionalized cations and anions respectively. The residues of **1** ~ **3** are molybdenum oxides.

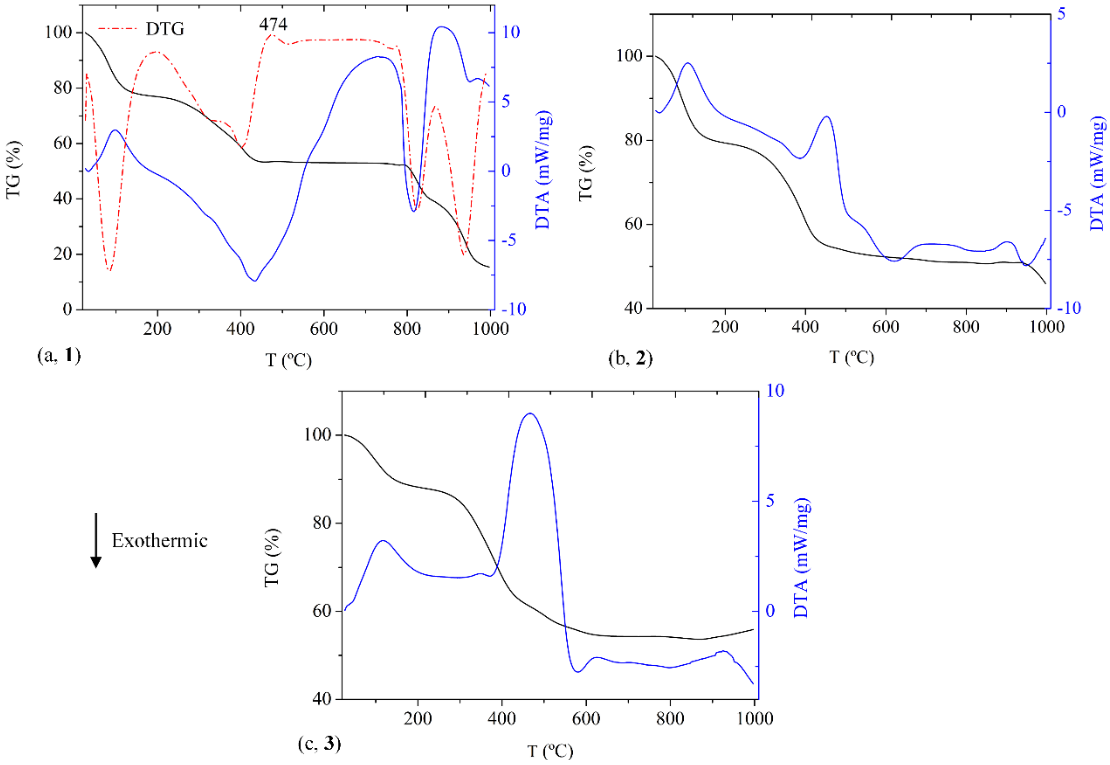

Supplementary Fig. 21. TG–DTA curves of $[\Delta/\Lambda\text{-}Mo^{*}{}_{2}O_{2}(\mu_{2}\text{-}S)(\mu_{2}\text{-}O)(R\text{-}Hlact^{*})_{2}(trz)_{2}(trz)]\cdot\frac{1}{2}H_{2}O$ (**1**, a), $[\Lambda/\Delta\text{-}Mo^{*}{}_{2}O_{2}(\mu_{2}\text{-}S)(\mu_{2}\text{-}O)(S\text{-}Hlact^{*})_{2}(trz)_{2}(trz)]$ (**2**, b) and $[Mo_{2}O_{2}(\mu_{2}\text{-}S)(\mu_{2}\text{-}O)(Hglyc)_{2}(trz)_{2}(H_{2}O)]$ (**3**, c).

Differential Thermal analyses. Thermal analysis (DTA/TG) was used to characterize thermal decomposition of the compounds and estimate melting point range.[28] The TG curve of compounds **1** ~ **3** indicates that those compounds do not melt and are stable up to 120 ℃, at which temperature they begin to decompose. The transformation from powders into homogeneous oxide phase corresponds to two endothermic peaks in the DTA curves of **1** ~ **3** which are shown in Supplementary Fig. 21. The first one around 120 ℃ is related to remove of tiny water and the weight losses of 1,2,4-triazoles. The second peaks at 474 ℃ in **1**, 452 ℃ in **2** and 467 ℃ in **3** corresponds to decomposition reactions: change into the corresponding molybdenum oxides. Therefore, the melting point of **1** ~ **3** (actually decomposed into oxides) are approximately 474 ℃, 452 ℃, 467 ℃ respectively. Among them, the endothermic

peak at 474 ℃ in **1** can clearly see from the corresponding DTG curve (red). Subsequent exothermic peaks may correspond to oxidation of evolved gas such as carbon monoxide.

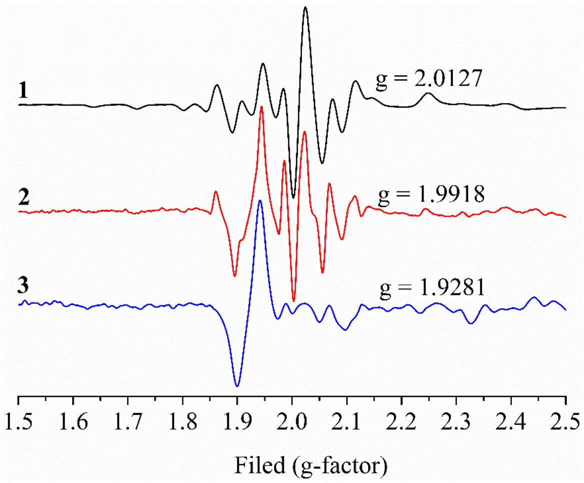

Supplementary Fig. 22. X-bands of EPR spectra for [Δ/Λ-Mo*$_2$O$_2$(μ$_2$-S)(μ$_2$-O)(*R*-Hlact*)$_2$(trz)$_2$(trz)]·½H$_2$O (**1**), [Λ/Δ-Mo*$_2$O$_2$(μ$_2$-S)(μ$_2$-O)(*S*-Hlact*)$_2$(trz)$_2$(trz)] (**2**) and [Mo$_2$O$_2$(μ$_2$-S)(μ$_2$-O)(Hglyc)$_2$(trz)$_2$(H$_2$O)] (**3**) in solid states at 90 K.

Theoretical bond valence calculations and EPR spectroscopy analyses. Theoretical bond valence calculations give the valences of 5.214_{av}, 5.206_{av}, 5.224_{av} for molybdenum atoms in **1** ~ **3** respectively, which can be seen in Supplementary Table 15. These are consistent with charge balance of +5 and further supported by electron paramagnetic resonance (EPR) experiment. As shown in Supplementary Fig. 22, the X-band EPR spectra of **1** ~ **3** exhibit S = ½ signals centered on g = 2.013, 1.992 and 1.928 at 90 K respectively, which are the feature of a $4d^1$ metal center Mo^{5+}.[29,30] Moreover, the spectra of **1** and **2** show well-resolved hyperfine structures determined by ^{95}Mo nuclear spin respectively.[31]

Supplementary Table 1. Crystallographic data and structural refinements for complexes [Δ/Λ-$Mo^{*}{}_2O_2(\mu_2\text{-}S)(\mu_2\text{-}O)$(*R*-Hlact*)$_2(trz)_2$(trz)]·½$H_2O$ (**1**), [Λ/Δ-$Mo^{*}{}_2O_2(\mu_2\text{-}S)(\mu_2\text{-}O)$(*S*-Hlact*)$_2(trz)_2$(trz)] (**2**), and [$Mo_2O_2(\mu_2\text{-}S)(\mu_2\text{-}O)$(Hglyc)$_2(trz)_2$($H_2O$)] (**3**).

	**1**	**2**	**3**
Empirical formula	$C_{12}H_{20}Mo_2N_9O_{9.5}S$	$C_{12}H_{19}Mo_2N_9O_9S$	$C_8H_{14}Mo_2N_6O_{10}S$
Formula weight	666.31	657.30	578.19
Temperature/K	100.01	100.00	193.0
Crystal system	monoclinic	monoclinic	monoclinic
Space group	$P\ 2_1$	$P\ 2_1$	*P* c
a/Å	12.3886(2)	12.3965(2)	10.9661(1)
b/Å	15.1481(2)	15.0742(3)	6.1069(6)
c/Å	12.4144(2)	12.4250(2)	13.3084(1)
α/ °	90	90	90
β/ °	103.7620(1)	103.832(2)	104.846(1)
γ/ °	90	90	90
Volume/Å^3	2262.85(6)	2254.50(7)	861.50(2)
Z	4	4	2
ρ_{calc}g/cm^3	1.956	1.937	2.229
μ/mm^{-1}	10.554	10.566	1.643
F(000)	1324.0	1304.0	568.0
Crystal size/mm^3	0.2 × 0.1 × 0.02	0.14 × 0.1 × 0.05	0.2 × 0.1 × 0.05
Radiation	CuKα (λ = 1.54184)	CuKα (λ = 1.54184)	MoKα (λ = 0.71073)
2*θ* range for data collection/ °	7.332 to 155.646	7.328 to 136.468	6.334 to 59.824
Reflections collected	9469	12325	3036

Independent reflections	9469	6835	3036
R_{sigma}	0.0297	0.0273	0.0735
Data/restraints/parameters	9469/22/618	6835/360/616	3036/11/251
Goodness of fit on F^2	1.072	1.030	1.027
Final R indexes [$I>=2\sigma (I)$]	R_1 = 0.0465, wR_2 = 0.1266	R_1 = 0.0275, wR_2 = 0.0741	R_1 = 0.0411, wR_2 = 0.0836
Final R indexes [all data]	R_1 = 0.0469, wR_2 = 0.1270	R_1 = 0.0280, wR_2 = 0.0744	R_1 = 0.0500, wR_2 = 0.0882
Largest diff. peak/hole / e Å^{-3}	1.67/-0.81	1.00/-0.91	1.05/-1.02
Flack parameter	0.010(7)	0.018(6)	-0.04(6)

Supplementary Table 2. Selected hydrogen bond distances (Å) and angles (°) in [Δ/Λ-Mo*$_2$O$_2$(μ$_2$-S)(μ$_2$-O)(*R*-Hlact*)$_2$(trz)$_2$(trz)]·½H_2O (**1**).

Donor–H ···Acceptor	D–H(Å)	H ···A(Å)	D ···A(Å)	D–H ···A(°)
O1–H1 ···N8	0.86(4)	2.53(4)	3.349(1)	161(3)
O1–H1 ···N9	0.86(4)	1.89(6)	2.617(1)	141(1)
O1w–H1w ···O2	0.85	2.14	2.79(3)	133
N3–H3 ···O6a	0.88	2	2.792(1)	149
N3–H3 ···O15b	0.88	2.59	3.164(2)	124
O4–H4 ···N8	0.95	1.78	2.718(1)	171
N6–H6 ···O3c	0.88	1.86	2.704(1)	161
N7–H7A ···O17b	0.88	1.87	2.666(1)	150
O10–H10A ···N18	0.85(8)	1.78(6)	2.574(2)	154(9)
N12–H12 ···O6	0.88	2.54	3.064(2)	119
N12–H12 ···O15c	0.88	1.95	2.787(1)	158
O13–H13 ···N17	0.85(3)	1.84(3)	2.682(1)	169(2)
N15–H15 ···O12a	0.88	1.89	2.703(1)	153
N16–H16 ···O8	0.88	1.9	2.684(1)	148

Symmetry codes: (a) 1 + *x*, *y*, *z*; (b) 1 + *x*, *y*, 1 + *z*; (c) *x*, *y*, 1 + *z*. The value in brackets represents the error.

Supplementary Table 3. Selected hydrogen bond distances (Å) and angles (°) in [Λ/Δ-$Mo^{*}_{2}O_{2}(\mu_2\text{-S})(\mu_2\text{-O})$(*S*-$Hlact^{*})_2(trz)_2$(trz)] (**2**).

Donor–H ···Acceptor	D–H	H ···A	D ···A	D–H ···A
O1–H1 ···N9	0.85(3)	1.88(3)	2.717(8)	169(3)
N3–H3 ···O6a	0.88	1.86	2.707(8)	160
O4–H4 ···N8	0.85(3)	1.76(3)	2.609(7)	174(4)
O4–H4 ···N9	0.85(3)	2.60(3)	3.345(7)	147(5)
N6–H6 ···O3b	0.88	2.02	2.820(7)	150
N6–H6 ···O12c	0.88	2.55	3.134(8)	125
N7–H7A ···O17c	0.88	1.9	2.700(7)	150
O10–H10A ···N18	0.85(2)	1.83(2)	2.681(8)	172(6)
N12–H12 ···O15b	0.88	1.88	2.701(8)	154
O13–H13 ···N17	0.86(1)	1.74(2)	2.566(9)	163(2)
N15–H15 ···O3	0.92(1)	2.52(1)	3.048(8)	117(9)
N15–H15 ···O12a	0.92(1)	1.93(1)	2.773(8)	151(1)
N16–H16 ···O8	0.88	1.91	2.686(7)	146

Symmetry codes: (a) *x*, *y*, -1 + *z*; (b) -1 + *x*, *y*, *z*; (c) -1 + *x*, *y*, -1 + *z*.

Supplementary Table 4. Selected hydrogen bond distances (Å) and angles (°) in $[Mo_2O_2(\mu_2\text{-}S)(\mu_2\text{-}O)(Hglyc)_2(trz)_2(H_2O)]$ (**3**).

Donor–H ···Acceptor	D–H	H ···A	D ···A	D–H ···A
O1–H1 ···O1wa	0.86(5)	1.84(6)	2.675(9)	165(6)
O1w–H1wA ···O8b	0.87	1.82	2.670(9)	166
O1w–H1wB ···O7	0.86	2.27	3.051(9)	150
N3–H3 ···O6c	0.88	2.01	2.766(1)	143
O4–H4 ···O1wa	0.86(6)	1.83(6)	2.675(9)	167(3)
N6–H6A ···O3d	0.88	1.97	2.830(1)	164

Symmetry codes: (a) *x*, 1 – *y*, -½ + *z*; (b) *x*, 2 – *y*, ½ + *z*; (c) 1 + *x*, 1 – *y*, ½ + *z*; (d) -1 + *x*, -1 + *y*, *z*.

Supplementary Table 5. Selected bond distances (Å) and angles (°) for [Δ/Λ-$Mo^{*}_{2}O_{2}(\mu_{2}$-S)(μ_{2}-O)(*R*-Hlact*)$_{2}$(trz)$_{2}$(trz)]·½H_2O (**1**).

**1**			
Mo2–Mo1	2.6752(9)	Mo4–Mo3	2.6975(1)
Mo2–S1	2.318(3)	Mo4–S2	2.330(3)
Mo2–O4	2.266(8)	Mo4–O13	2.221(9)
Mo2–O5	2.113(7)	Mo4–O17	1.979(8)
Mo2–O8	1.924(6)	Mo4–O14	2.115(7)
Mo2–N4	2.231(9)	Mo4–N13	2.207(9)
Mo2–O9	1.678(9)	Mo4–O18	1.660(1)
Mo1–S1	2.321(2)	Mo3–S2	2.323(3)
Mo1–O2	2.123(7)	Mo3–O16	1.660(1)
Mo1–O8	1.947(7)	Mo3–O10	2.234(9)
Mo1–O1	2.251(9)	Mo3–O11	2.129(7)
Mo1–O7	1.690(9)	Mo3–O17	1.951(7)
Mo1–N1	2.224(8)	Mo3–N10	2.227(9)
S1–Mo2–Mo1	54.84(6)	O14–Mo4–O13	71.8(3)
O4–Mo2–Mo1	98.3(2)	O14–Mo4–N13	83.8(3)
O4–Mo2–S1	87.0(2)	N13–Mo4–Mo3	144.0(2)
O5–Mo2–Mo1	129.4(2)	N13–Mo4–S2	89.6(2)
O5–Mo2–S1	157.0(2)	N13–Mo4–O13	76.2(4)
O5–Mo2–O4	70.2(3)	O18–Mo4–Mo3	103.3(4)
O5–Mo2–N4	83.5(3)	O18–Mo4–S2	106.6(4)
O8–Mo2–Mo1	46.63(2)	O18–Mo4–O13	158.8(4)
O8–Mo2–S1	99.3(2)	O18–Mo4–O17	105.5(4)
O8–Mo2–O4	87.9(3)	O18–Mo4–O14	93.8(4)
O8–Mo2–O5	83.1(3)	O18–Mo4–N13	87.0(4)
O8–Mo2–N4	162.7(3)	S2–Mo3–Mo4	54.69(7)
N4–Mo2–Mo1	143.8(2)	O16–Mo3–Mo4	101.8(3)
N4–Mo2–S1	89.0(2)	O16–Mo3–S2	106.3(3)
N4–Mo2–O4	77.2(3)	O16–Mo3–O10	160.1(4)
O9–Mo2–Mo1	100.7(3)	O16–Mo3–O11	93.1(4)
O9–Mo2–S1	106.1(3)	O16–Mo3–O17	104.0(4)
O9–Mo2–O4	160.8(3)	O16–Mo3–N10	86.8(4)
O9–Mo2–O5	95.5(4)	O10–Mo3–Mo4	97.9(2)
O9–Mo2–O8	103.3(4)	O10–Mo3–S2	87.8(2)
O9–Mo2–N4	88.8(4)	O11–Mo3–Mo4	130.6(2)
S1–Mo1–Mo2	54.74(6)	O11–Mo3–S2	158.7(3)
O2–Mo1–Mo2	129.5(2)	O11–Mo3–O10	71.3(3)
O2–Mo1–S1	157.4(2)	O11–Mo3–N10	84.3(3)
O2–Mo1–O1	70.6(3)	O17–Mo3–Mo4	47.1(2)
O2–Mo1–N1	83.9(3)	O17–Mo3–S2	99.7(2)

O8–Mo1–Mo2	45.93(2)	O17–Mo3–O10	87.0(4)
O8–Mo1–S1	98.5(2)	O17–Mo3–O11	83.7(3)
O8–Mo1–O2	83.9(3)	O17–Mo3–N10	164.3(4)
O8–Mo1–O1	85.7(3)	N10–Mo3–Mo4	142.6(2)
O8–Mo1–N1	162.8(4)	N10–Mo3–S2	88.0(2)
O1–Mo1–Mo2	96.4(2)	N10–Mo3–O10	79.5(4)
O1–Mo1–S1	87.1(2)	Mo2–S1–Mo1	70.42(7)
O7–Mo1–Mo2	100.2(3)	Mo3–S2–Mo4	70.86(8)
O7–Mo1–S1	105.5(3)	C1–O2–Mo1	125.1(7)
O7–Mo1–O2	95.7(4)	C5–O4–Mo2	118.9(7)
O7–Mo1–O8	103.4(4)	C14–O10–Mo3	119.2(7)
O7–Mo1–O1	162.9(3)	C4–O5–Mo2	124.8(7)
O7–Mo1–N1	90.0(4)	C17–O13–Mo4	117.2(7)
N1–Mo1–Mo2	142.9(2)	C13–O11–Mo3	123.2(7)
N1–Mo1–S1	88.2(2)	Mo2–O8–Mo1	87.4(3)
N1–Mo1–O1	78.8(3)	Mo3–O17–Mo4	86.7(3)
S2–Mo4–Mo3	54.45(7)	C16–O14–Mo4	121.9(8)
O13–Mo4–Mo3	97.9(3)	C2–O1–Mo1	120.6(6)
O13–Mo4–S2	86.5(2)	C21–N13–Mo4	124.7(8)
O17–Mo4–Mo3	46.2(2)	C22–N13–Mo4	133.5(7)
O17–Mo4–S2	98.6(2)	C9–N4–Mo2	125.2(7)
O17–Mo4–O13	88.5(4)	C10–N4–Mo2	129.6(8)
O17–Mo4–O14	82.8(3)	C7–N1–Mo1	126.1(8)
O17–Mo4–N13	162.2(4)	C8–N1–Mo1	130.7(8)
O14–Mo4–Mo3	128.8(2)	C20–N10–Mo3	132.4(8)
O14–Mo4–S2	158.2(3)	C19–N10–Mo3	125.7(8)

The value in brackets represents the error.

Supplementary Table 6. Selected bond distances (Å) and angles (°) for [Λ/Δ-$Mo^{*}_2O_2(\mu_2$-S)(μ_2-O)(*S*-$Hlact^{*})_2(trz)_2$(trz)] (**2**).

**2**			
Mo2–Mo1	2.6719(6)	Mo4–Mo3	2.6961(6)
Mo2–S1	2.3248(1)	Mo4–S2	2.3245(2)
Mo2–O4	2.247(5)	Mo4–O18	1.679(5)
Mo2–O5	2.129(4)	Mo4–O13	2.236(6)
Mo2–O8	1.938(4)	Mo4–O17	1.940(4)
Mo2–O9	1.678(5)	Mo4–O14	2.135(4)
Mo2–N4	2.232(5)	Mo4–N13	2.238(5)
Mo1–S1	2.3240(2)	Mo3–S2	2.3341(2)
Mo1–O1	2.263(5)	Mo3–O10	2.236(5)
Mo1–O2	2.114(4)	Mo3–O11	2.122(4)
Mo1–O8	1.933(4)	Mo3–O17	1.945(4)
Mo1–O7	1.672(5)	Mo3–O16	1.664(5)
Mo1–N1	2.220(5)	Mo3–N10	2.221(5)
S1–Mo2–Mo1	54.90(4)	O17–Mo4–Mo3	46.15(1)
O4–Mo2–Mo1	96.72(1)	O17–Mo4–S2	98.85(1)
O4–Mo2–S1	87.01(1)	O17–Mo4–O13	87.2(2)
O5–Mo2–Mo1	129.69(1)	O17–Mo4–O14	84.62(2)
O5–Mo2–S1	157.51(1)	O17–Mo4–N13	164.9(2)
O5–Mo2–O4	70.83(2)	O14–Mo4–Mo3	130.56(1)
O5–Mo2–N4	84.08(2)	O14–Mo4–S2	158.40(2)
O8–Mo2–Mo1	46.27(1)	O14–Mo4–O13	71.06(2)
O8–Mo2–S1	98.74(1)	O14–Mo4–N13	84.13(2)
O8–Mo2–O4	85.24(2)	N13–Mo4–Mo3	142.87(1)
O8–Mo2–O5	83.61(2)	N13–Mo4–S2	88.08(1)
O8–Mo2–N4	162.4(2)	S2–Mo3–Mo4	54.47(4)
O9–Mo2–Mo1	99.70(2)	O10–Mo3–Mo4	98.80(1)
O9–Mo2–S1	105.20(2)	O10–Mo3–S2	86.83(1)
O9–Mo2–O4	163.24(2)	O11–Mo3–Mo4	128.63(1)
O9–Mo2–O5	95.8(2)	O11–Mo3–S2	158.14(2)
O9–Mo2–O8	103.8(2)	O11–Mo3–O10	71.34(2)
O9–Mo2–N4	90.0(2)	O11–Mo3–N10	83.89(2)
N4–Mo2–Mo1	142.88(1)	O17–Mo3–Mo4	45.98(1)
N4–Mo2–S1	87.98(1)	O17–Mo3–S2	98.37(1)
N4–Mo2–O4	78.8(2)	O17–Mo3–O10	89.1(2)
S1–Mo1–Mo2	54.93(4)	O17–Mo3–O11	82.89(2)
O1–Mo1–Mo2	98.05(1)	O17–Mo3–N10	162.4(2)
O1–Mo1–S1	86.42(1)	O16–Mo3–Mo4	103.36(2)
O2–Mo1–Mo2	129.42(1)	O16–Mo3–S2	106.52(2)
O2–Mo1–S1	156.67(1)	O16–Mo3–O10	157.8(2)

O2–Mo1–O1	70.42(2)	O16–Mo3–O11	94.0(2)
O2–Mo1–N1	83.68(2)	O16–Mo3–O17	105.8(2)
O8–Mo1–Mo2	46.43(1)	O16–Mo3–N10	86.7(3)
O8–Mo1–S1	98.92(1)	N10–Mo3–Mo4	144.10(1)
O8–Mo1–O1	87.33(2)	N10–Mo3–S2	89.65(1)
O8–Mo1–O2	83.25(2)	N10–Mo3–O10	75.6(2)
O8–Mo1–N1	162.6(2)	Mo1–S1–Mo2	70.16(4)
O7–Mo1–Mo2	100.40(2)	Mo4–S2–Mo3	70.72(4)
O7–Mo1–S1	105.80(2)	C2–O1–Mo1	119.0(4)
O7–Mo1–O1	161.45(2)	C1–O2–Mo1	124.2(4)
O7–Mo1–O2	96.1(2)	C5–O4–Mo2	120.4(4)
O7–Mo1–O8	104.1(2)	C14–O10–Mo3	118.3(4)
O7–Mo1–N1	88.7(2)	C17–O13–Mo4	119.6(4)
N1–Mo1–Mo2	143.58(1)	C4–O5–Mo2	124.0(4)
N1–Mo1–S1	88.65(2)	C13–O11–Mo3	121.8(4)
N1–Mo1–O1	77.4(2)	Mo1–O8–Mo2	87.31(2)
S2–Mo4–Mo3	54.80(4)	Mo4–O17–Mo3	87.88(2)
O18–Mo4–Mo3	101.76(2)	C16–O14–Mo4	123.4(4)
O18–Mo4–S2	106.67(2)	C8–N1–Mo1	130.2(5)
O18–Mo4–O13	160.0(2)	C7–N1–Mo1	125.2(4)
O18–Mo4–O17	103.8(2)	C20–N10–Mo3	131.9(5)
O18–Mo4–O14	93.0(2)	C19–N10–Mo3	123.4(4)
O18–Mo4–N13	86.8(2)	C9–N4–Mo2	125.2(4)
O13–Mo4–Mo3	97.93(1)	C10–N4–Mo2	131.1(5)
O13–Mo4–S2	87.75(1)	C22–N13–Mo4	131.1(5)
O13–Mo4–N13	79.7(2)	C21–N13–Mo4	124.8(5)

Supplementary Table 7. Selected bond distances (Å) and angles (°) for $[Mo_2O_2(\mu_2\text{-}S)(\mu_2\text{-}O)(Hglyc)_2(trz)_2(H_2O)]$ (**3**).

**3**			
Mo1–Mo2	2.6672(9)	Mo2–S1	2.335(3)
Mo1–S1	2.324(2)	Mo2–O8	1.946(6)
Mo1–O8	1.932(7)	Mo2–O4	2.285(7)
Mo1–O1	2.282(6)	Mo2–N4	2.216(8)
Mo1–O7	1.679(6)	Mo2–O5	2.083(7)
Mo1–O2	2.092(6)	Mo2–O9	1.680(7)
Mo1–N1	2.191(8)		
S1–Mo1–Mo2	55.28(7)	O8–Mo2–O5	84.9(3)
O8–Mo1–Mo2	46.77(2)	O4–Mo2–Mo1	99.79(2)
O8–Mo1–S1	98.7(2)	O4–Mo2–S1	86.00(2)
O8–Mo1–O1	85.1(3)	N4–Mo2–Mo1	143.3(2)
O8–Mo1–O2	85.0(3)	N4–Mo2–S1	88.5(2)
O8–Mo1–N1	162.2(3)	N4–Mo2–O4	77.6(3)
O1–Mo1–Mo2	98.18(2)	O5–Mo2–Mo1	131.17(2)
O1–Mo1–S1	86.04(2)	O5–Mo2–S1	156.61(2)
O7–Mo1–Mo2	98.5(2)	O5–Mo2–O4	70.9(2)
O7–Mo1–S1	105.8(2)	O5–Mo2–N4	83.2(3)
O7–Mo1–O8	104.5(3)	O9–Mo2–Mo1	97.8(2)
O7–Mo1–O1	163.1(3)	O9–Mo2–S1	105.2(3)
O7–Mo1–O2	95.8(3)	O9–Mo2–O8	104.2(3)
O7–Mo1–N1	89.6(3)	O9–Mo2–O4	162.4(3)
O2–Mo1–Mo2	131.7(2)	O9–Mo2–N4	89.0(3)
O2–Mo1–S1	156.35(2)	O9–Mo2–O5	96.5(3)
O2–Mo1–O1	70.9(2)	Mo1–S1–Mo2	69.83(7)
O2–Mo1–N1	82.9(3)	Mo1–O8–Mo2	86.9(3)
N1–Mo1–Mo2	142.8(2)	C2–O1–Mo1	116.7(5)
N1–Mo1–S1	87.5(2)	C4–O4–Mo2	118.3(5)
N1–Mo1–O1	78.7(3)	C1–O2–Mo1	125.7(6)
S1–Mo2–Mo1	54.89(6)	C8–N4–Mo2	133.9(6)
O8–Mo2–Mo1	46.3(2)	C7–N4–Mo2	122.4(7)
O8–Mo2–S1	97.9(2)	C3–O5–Mo2	126.4(6)
O8–Mo2–O4	87.3(3)	C6–N1–Mo1	128.7(7)
O8–Mo2–N4	163.2(3)	C5–N1–Mo1	127.2(7)

Supplementary Table 8. Detail calibrated adsorption data of O_2, N_2, H_2, CO_2 and CH_4 for [Λ/Δ-$Mo^{*}_2O_2$(μ_2-S)(μ_2-O)(*S*-$Hlact^{*}$)$_2$(trz)$_2$(trz)] (**2**).

Gases	O_2		CO_2		CH_4		N_2		H_2	
Temperature (ºC)	Pressure (bar)	Adsorption (mg/g)	Pressure (bar)	Adsorption (mg/g)	Pressure (bar)	Adsorption (mg/g)	Pressure (bar)	Adsorption (mg/g)	Pressure (bar)	Adsorption (mg/g)
25.0	0	0	0	0	0	0	0	0	0	0
	1.890	1.7193	1.900	2.2802	1.900	0.3396	1.900	0.4301	1.920	0.1173
	3.894	2.9943	3.895	3.4581	3.900	0.4503	3.900	0.4771	3.900	0.0577
	5.900	4.2824	5.898	4.6204	5.898	0.4415	5.894	0.5740	5.897	0.0343
	7.897	5.6792	7.893	5.5768	7.896	0.4854	7.897	0.6348	7.902	0.1136
	9.897	6.9754	9.893	6.3254	9.896	0.5086	9.894	0.6016	9.903	0.0728
	11.897	8.0490	11.895	6.9945	11.897	0.4925	11.896	0.5992	11.911	0.0138
	13.896	9.2541	13.894	7.5706	13.899	0.5568	13.898	0.5114	13.908	0.0495
	15.893	10.4207	15.894	8.3032	15.894	0.6907	15.899	0.5800	15.910	0.0922
	17.896	11.7313	17.891	8.7941	17.898	0.6454	17.896	0.5411	17.907	0.0674
	19.895	12.8728	19.892	9.2422	19.896	0.5239	19.896	0.4152	19.909	0.0495
	21.896	14.1802	21.892	9.6216	21.898	0.4576	21.894	0.0658	21.904	0.1294
	23.897	15.4770	23.893	10.0777	23.898	0.3479	23.896	0.4547	23.908	0.1393
	25.896	16.6082	25.891	10.2523	25.897	0.3276	25.897	0.7008	25.904	0.1729
	27.896	17.6086	27.890	10.6862	27.897	0.2442	27.897	0.7190	27.908	0.1422
	29.894	19.2340	29.891	10.8411	29.897	0.2638	29.894	0.1340	29.905	0.1361

Supplementary Table 9. Detail calibrated adsorption data of O_2 and N_2 for [Δ/Λ-$Mo^{*}_2O_2(\mu_2$-S)(μ_2-O)(*R*-Hlact*)$_2$(trz)$_2$(trz)]·½H_2O (**1**).

Gases	O_2		N_2	
Temperature (°C)	Pressure (bar)	Adsorption (mg/g)	Pressure (bar)	Adsorption (mg/g)
25.0	0	0	0	0
	1.890	1.4455	1.900	0.1980
	3.898	2.6793	3.890	0.2921
	5.894	3.8940	5.891	0.1725
	7.895	5.1682	7.900	0.2788
	9.897	6.0633	9.897	0.7341
	11.897	7.1437	11.896	0.3540
	13.894	8.5547	13.900	0.2062
	15.896	9.7397	15.897	0.2879
	17.895	10.8941	17.893	0.2162
	19.896	11.7403	19.896	0.2596
	21.896	13.1430	21.894	0.1519
	23.892	14.4176	23.894	0.0774
	25.893	15.2043	25.896	0.2977
	27.895	16.6807	27.894	0.3849
	29.895	17.6979	29.895	0.1193

Supplementary Table 10. Comparisons of selected bond distances (Å) for [Δ/Λ-$Mo^*{}_2O_2$(μ_2-S)(μ_2-O)(*R*-$Hlact^*$)$_2$(trz)$_2$(trz)]·½H_2O (**1**), [Λ/Δ-$Mo^*{}_2O_2$(μ_2-S)(μ_2-O)(*S*-$Hlact^*$)$_2$(trz)$_2$(trz)] (**2**), [Mo_2O_2(μ_2-S)(μ_2-O)(Hglyc)$_2$(trz)$_2$(H_2O)] (**3**), (PyH)$_2$[Mo_2O_4(glyc)$_2$Py$_2$] (**4**),[1] (PyH)$_4$[$Mo_4O_8Cl_4$(glyc)$_2$] ·2EtOH (**5**),[1] [Mo_4O_8(glyc)$_2$Py$_4$] (**6**),[1] K_6[(MoO_2)$_8$(glyc)$_6$(Hglyc)$_2$] ·10H_2O (**7**),[2] *trans*-[(MoO)$_2$O(glyc)$_2$(bpy)$_2$] ·3H_2O (**8**),[3] *trans*-[(MoO)$_2$O(*R*,*S*-lact)$_2$(bpy)$_2$] ·3H_2O (**9**),[3] *trans*-[(MoO)$_2$O(*R*,*S*-lact)$_2$(phen)$_2$] ·4H_2O (**10**),[3] Na_2[Mo_3(μ_3-S)(μ_2-O)$_3$(glyc)$_3$(2-mim)$_3$] ·1.5H_2O (**11**),[4] (4-Hmim)$_6$[Mo_3(μ_3-S)(μ_2-O)$_3$(glyc)$_3$(4-mim)$_3$]$_2$[MoO_2(glyc)$_2$] (**12**),[4] Na_3(4-Hmim)[Mo_3(μ_3-S)(μ_2-O)$_3$(SO_3)(glyc)$_3$(4-mim)] ·8H_2O (**13**),[4] [Mo_3SO_3(glyc)$_2$(im)$_5$] ·im ·H_2O (**14**),[5] Na_2[Mo_3SO_3(*R*,*S*-lact)$_3$(im)$_3$] ·10H_2O (**15**),[5] [Mo_3S_4(PPh_3)$_3$(Hlact)$_2$(lact)] (**16**),[6] K_2[MoO_2(glyc)$_2$] ·H_2O (**17**),[2] [(C6H5)4P][MoO2(glyc)(Hglyc)] (**18**),[1] [Na_2[MoO_2(*S*-lact)$_2$]]$_3$ ·13H_2O (**19**).[2]

Complexes	Mo–$O_{\alpha\text{-hydroxy}}$	Mo–$O_{\alpha\text{-carboxy}}$	C–$O_{\alpha\text{-hydroxy}}$
Mo(V)			
**1**	2.251(9)	2.123(7)	1.426(1)
	2.266(8)	2.113(7)	1.390(2)
	2.221(9)	2.115(7)	1.433(1)
	2.234(9)	2.129(7)	1.427(1)
**2**	2.236(5)	2.122(4)	1.418(8)
	2.236(6)	2.135(4)	1.436(8)
	2.247(5)	2.129(4)	1.425(7)
	2.263(5)	2.114(4)	1.413(8)
**3**	2.285(7)	2.083(7)	1.418(1)
	2.282(6)	2.092(6)	1.443(1)
average	2.252(7)	2.116(6)	1.423(4)
	Mo–$O_{\alpha\text{-alkoxy}}$	Mo–$O_{\alpha\text{-carboxy}}$	C–$O_{\alpha\text{-alkoxy}}$
**4**	2.183(2)	2.092(2)	1.420(3)
**5**	2.245(3)/2.115(2)	2.117(3)	1.424(5)
**6**	2.183(3)/2.097(3)	2.046(3)	1.419(5)
**7**	1.984(7)<br>2.073(5)/2.182(5)<br>2.130(5)/2.089(5)	$2.064(6)_{av}$	$1.408(1)_{av}$
average	2.122(4)	2.076(4)	1.417(4)
**8**	$1.958(3)_{av}$	$2.067(4)_{av}$	$1.417(6)_{av}$
**9**	$1.953(6)_{av}$	$2.051(7)_{av}$	$1.415(1)_{av}$
**10**	$1.958(2)_{av}$	$2.064(2)_{av}$	$1.405(4)_{av}$
average	1.956(4)	2.061(5)	1.412(4)
	Mo–$O_{\alpha\text{-hydroxy/hydroxy}}$	Mo–$O_{\alpha\text{-carboxy}}$	C–$O_{\alpha\text{-hydroxy/hydroxy}}$
Mo(IV)			
**11**	$2.003(5)_{av}$	$2.172(6)_{av}$	$1.409(1)_{av}$
**12**	$2.011(3)_{av}$	$2.138(4)_{av}$	$1.414(7)_{av}$
**13**	$2.016(2)_{av}$	$2.143(2)_{av}$	$1.412(4)_{av}$
**14**	$1.986(2)_{av}$	$2.127(2)_{av}$	$1.397(5)_{av}$

**15**	$1.999(7)_{av}$	$2.133(6)_{av}$	$1.42(2)_{av}$
**16**	$2.092(3)/2.204(4)_{av}$	$2.083(4)/2.118(4)_{av}$	$1.299(7)/1.422(8)_{av}$
average	2.018(4)/2.204(4)	2.133(4)/2.118(4)	1.392(4)/1.422(8)
Mo(VI)			
**17**	$1.941(2)_{av}$	$2.203(3)_{av}$	$1.404(5)_{av}$
**18**	1.945(1)	2.271(1)	1.392(2)
**19**	$1.958(5)_{av}$	$2.191(5)_{av}$	$1.425(8)_{av}$
average	1.948(3)	2.222(3)	1.407(5)

Supplementary Table 11. The bond distances (Å) of Mo–O$_{\alpha\text{-alkoxy/hydroxy}}$, Mo–O$_{\alpha\text{-carboxy}}$ and C–O$_{\alpha\text{-alkoxy/hydroxy}}$ in FeMo-cos of nitrogenases (**20**).

PDB ID	Resolutions	Mo–O$_{\alpha\text{-alkoxy}/\alpha\text{-hydroxy}}$	Mo–O$_{\alpha\text{-carboxy}}$	C–O$_{\alpha\text{-alkoxy}/\alpha\text{-hydroxy}}$
36 structures of FeMo-cofactors[5]		2.272$_{av}$	2.263$_{av}$	1.449$_{av}$
6CDK[32]	2.1 Å	1.949, 1.936	1.989, 1.966	1.449, 1.451
6O7L[33]	2.26 Å	2.492, 2.367	2.371, 2.459	1.433, 1.432
6O7M[33]	1.4 Å	2.187, 2.241	2.208, 2.194	1.437, 1.436
6O7N[33]	1.75 Å	2.211, 2.299	2.275, 2.194	1.425, 1.442
6O7O[33]	1.89 Å	2.231, 2.268	2.249, 2.244	1.442, 1.430
6O7P[33]	1.7 Å	2.340, 2.295	2.272, 2.290	1.435, 1.445
6O7Q[33]	2 Å	2.390, 2.377	2.386, 2.366	1.435, 1.437
6O7R[33]	2.27 Å	2.303, 2.371	2.421, 1.765	1.440, 1.438
6O7S[33]	2.27 Å	2.389, 2.324	2.438, 2.419	1.430, 1.427
6OP1[34]	1.7 Å	2.261, 2.243	2.235, 2.204	1.439, 1.446
6OP2[34]	1.9 Å	2.274,2.002	2.212, 2.156	1.450, 1.446
6OP3[34]	1.6 Å	2.216, 2.215	2.172, 2.174	1.436, 1.439
6OP4[34]	2.3 Å	2.137, 2.220	2.217, 2.184	1.446, 1.450
Average		2.263	2.250	1.445

Bond distances comparison. Comparisons of selected bond distances (Å) for oxidomolybdenum(V) lactates and glycolates **1** ~ **3** and the other molybdenum(IV/V/VI) lactates, glycolates **4** ~ **19** are shown in Supplementary Tables 5 ~ 7 and 10 ~ 11 respectively. The protonated Mo(V)–O$_{\alpha\text{-hydroxy}}$ distances [2.252(7)$_{av}$ Å] in **1** ~ **3** are obviously longer than Mo(V)–O$_{\alpha\text{-alkoxy}}$ distances [2.183(2) Å] in **4** in identical coordination mode, and longer than Mo(V)–O$_{\alpha\text{-alkoxy}}$ distances in **5** ~ **7** [2.122(4)$_{av}$ Å] in multi-cores of oxidomolybdenum glycolates. We can also found that Mo–O$_{\alpha\text{-alkoxy}}$ distances [1.958(3)$_{av}$ Å] in **8**, [1.956(4)$_{av}$ Å] in **9** ~ **10** are significantly reduced in bridged monooxy coordination mode compared with other coordination pattern simultaneously. Similarly, we can observe the protonated Mo(IV)–O$_{\alpha\text{-hydroxy}}$ distances in **16** [2.204(4)$_{av}$ Å] are longer than Mo(IV)–O$_{\alpha\text{-alkoxy}}$ distances in **11** ~ **16** [2.018(4)$_{av}$ Å], this indicates that protonation can lengthen the Mo–O distance and is common over 2.2 Å. The Mo(IV)–O$_{\alpha\text{-alkoxy}}$ distances in **11** ~ **16** [2.018(4)$_{av}$ Å] are

longer than Mo(V)–$O_{\alpha\text{-alkoxy}}$ distances in **8** ~ **10** [1.956(4)$_{av}$ Å] and Mo(VI)–$O_{\alpha\text{-alkoxy}}$ distances in **17** ~ **19** [1.948(3)$_{av}$ Å], this implied that the lower valence state of the metal Mo resulted in longer Mo–$O_{\alpha\text{-alkoxy}}$ bond distances. When $O_{\alpha\text{-alkoxy}}$ coordinated with two molybdenum atoms, the Mo–$O_{\alpha\text{-alkoxy}}$ distances increased (**4** ~ **7**). The protonated Mo(V)–$O_{\alpha\text{-hydroxy}}$ distances [2.252(7)$_{av}$ Å] are closer to the average value of Mo–O distances in FeMo-cofactors (**20**) [2.263$_{av}$ Å] of FeMo-proteins, as obtained from protein data bank in Supplementary Table 11. This supports that the oxygen atom in Mo–$O_{\alpha\text{-alkoxy}}$ should be protonated in FeMo-co.

The Mo(V)–$O_{\alpha\text{-carboxy}}$ distances [2.116(6)$_{av}$ Å] observed in **1** ~ **3** are longer than those of molybdenum(V) complexes **4** [2.092(2) Å], **5** ~ **7** [2.076(4)$_{av}$ Å], **8** ~ **10** [2.061(4)$_{av}$ Å], these are mainly attributed to the effects of protonation. The Mo(IV)–$O_{\alpha\text{-carboxy}}$ distances [2.133(4)$_{av}$ Å] in complexes **11** ~ **16** were longer than those [2.092(2)$_{av}$ Å] in molybdenum (V) complexes **4**, **5** ~ **7** [2.076(4)$_{av}$ Å] and **8** ~ **10** [2.061(5)$_{av}$ Å] owing to the influence of the metal valence, but shorter than those in **17** ~ **19** [2.222(3)$_{av}$ Å]. This might be due to the *trans*-effect. Unlike the Mo–$O_{\alpha\text{-alkoxy/hydroxy}}$ bond distances, the Mo–$O_{\alpha\text{-carboxy}}$ distances [2.116(6)$_{av}$ Å] showed no major difference to the average value of FeMo-cofactors [**20**, 2.250 Å].

Furthermore, the C–$O_{\alpha\text{-hydroxy}}$ distances [1.423(4)$_{av}$ Å] in **1** ~ **3** are little longer than complexes **5** ~ **7** [1.417(4)$_{av}$ Å], **8** ~ **10** [1.412(4)$_{av}$ Å], but closer to protonated **16** [1.422(8) Å], and significantly longer than Mo(IV) complexes **11** ~ **16** [1.392(4)$_{av}$ Å], Mo(VI) complexes **17** ~ **19** [1.407(5)$_{av}$ Å], loser to the average value in FeMo-cos [**20**, 1.445$_{av}$ Å]. So the Mo(IV/V/VI)–$O_{\alpha\text{-alkoxy/hydroxy}}$ bond is greatly affected by protonation and affect the neighboring C–O bond strength. Excluding the influence of oxidation state(Mo), the Mo(V)–$O_{\alpha\text{-hydroxy}}$, Mo(V)–$O_{\alpha\text{-carboxy}}$, and C–$O_{\alpha\text{-hydroxy}}$ distances in **1** ~ **3** all closer to FeMo-cos, supporting the fact that the oxygen atom

involved in Mo–$O_{\alpha\text{-alkoxy}}$ coordination in FeMo-co should be protonated.

Supplementary Table 12. Qualitative assignments of the most intensive bands in VCD spectra of FeMo-co **20** and $K_5[\Lambda,\Lambda,\Lambda,\Lambda\text{-}Mo^*_4O_{11}(R\text{-}Hhomocit^*)_2]Cl\cdot5H_2O$ **21**.

**20**	frequency assignments (cm^{-1})	**21**	frequency assignments (cm^{-1})
1657	$\nu_{as}(CO_2^-)$	1705	$\nu_{as}(\gamma\text{-}CO_2H)$
		1616	$\nu_{as}(CO_2^-)$
1622	$\nu_{as}(CO_2^-)$		
1608	ν_{as}(–NHCO)		
1527	NMF		
1479, 1383	$\nu_s(CO_2^-)$	1417, 1396	$\nu_s(CO_2^-)$
1327	ν_s(–NHCO)		
1429	NMF		
1271	NMF		
1189	ν(C–C)	1180	ν(C–C)
1153	ν(C–C)	1157	ν(C–C)
1068	$\nu(C\text{–}O_H)$	1084	ν(C–O)
957	–	953	ν(Mo=O)

Asymmetric and symmetric vibrations have been abbreviated as ν_{as} and ν_s respectively.

Supplementary Table 13. Comparison of obtained experimental IR, VCD charactermatic signals of [Δ/Λ-$Mo^*_2O_2$(μ_2-S)(μ_2-O)(*R*-$Hlact^*$)$_2$(trz)$_2$(trz)]·½H_2O (**1**) in the solid state and assignments of calculated VCD signals from optimized structure of **1**.

**1**	IR frequency (cm^{-1})	Experimental frequency (cm^{-1})	Theoretical frequency (cm^{-1})
ν_{as}(COO)	1641	1653	1672
ν_s(COO)	1379	1371	1352
ν(Mo=O)	960 ~ 976	959 ~ 976	989
ν(C–N)	1148 ~ 1171	1155 ~ 1180	1202 ~ 1213
ν(C–O_H)	1053	1051	1054

Supplementary Table 14. Comparisons of obtained experimental IR, VCD charactermatic signals of extracted FeMo-co Δ-Mo*Fe_7S_9C[*R*-(H)homocit*]$(NMF)_2$ (**20**) and assignments of calculated VCD signals from optimized structure of **20**.

**20**	IR frequency (cm^{-1})	Experimental frequency (cm^{-1})	Theoretical frequency (cm^{-1})
$\nu_{as}(CO_2^-)$	1674, 1608	1657, 1622	1655
$\nu_s(CO_2^-)$	1483, 1400	1479, 1383	1463, 1376
NMF	1425	1429	1412
ν(C–C)	1184	1189	1178
ν(C–C)	1163	1153	1165
$\nu(C–O_H)$	1063	1068	1058
–	984	957	945

Supplementary Table 15. Bond valence calculations for complexes [Δ/Λ-$Mo^{*}_2O_2$(μ_2-S)(μ_2-O)(*R*-$Hlact^{*}$)$_2$(trz)$_2$(trz)]·½H_2O (**1**), [Λ/Δ-$Mo^{*}_2O_2$(μ_2-S)(μ_2-O)(*S*-$Hlact^{*}$)$_2$(trz)$_2$(trz)] (**2**), and [Mo_2O_2(μ_2-S)(μ_2-O)(Hglyc)$_2$(trz)$_2$(H_2O)] (**3**).

Complexes	Atoms	N	$\sum S_{ij}$	Δ
[Δ/Λ-$Mo^{*}_2O_2$(μ_2-S)(μ_2-O)(*R*-$Hlact^{*}$)$_2$(trz)$_2$(trz)]·½H_2O (**1**)	Mo(1)	5+	5.122	0.122
	Mo(2)	5+	5.229	0.229
	Mo(3)	5+	5.267	0.267
	Mo(4)	5+	5.239	0.239
	**Mo_{av}**	**5+**	**5.214_{av}**	**0.214_{av}**
[Λ/Δ-$Mo^{*}_2O_2$(μ_2-S)(μ_2-O)(*S*-$Hlact^{*}$)$_2$(trz)$_2$(trz)] (**2**)	Mo(1)	5+	5.243	0.243
	Mo(2)	5+	5.169	0.169
	Mo(3)	5+	5.249	0.249
	Mo(4)	5+	5.164	0.164
	**Mo_{av}**	**5+**	**5.206_{av}**	**0.206_{av}**
[Mo_2O_2(μ_2-S)(μ_2-O)(Hglyc)$_2$(trz)$_2$(H_2O)] (**3**)	Mo(1)	5+	5.276	0.276
	Mo(2)	5+	5.172	0.172
	**Mo_{av}**	**5+**	**5.224_{av}**	**0.224_{av}**

Average has been abbreviated as av, bold represents the average result.

Part II IR spectra of 1,2,4-triazole, lactic acid, *N*-methylformamide, sodium homocitrate, $K_2[Mo^{VI}O_2(R,S\text{-}H_2homocit)_2]\cdot 2H_2O$, and $Na_2[Mo_3SO_3(R,S\text{-}lact)_3(im)_3]\cdot 10H_2O$

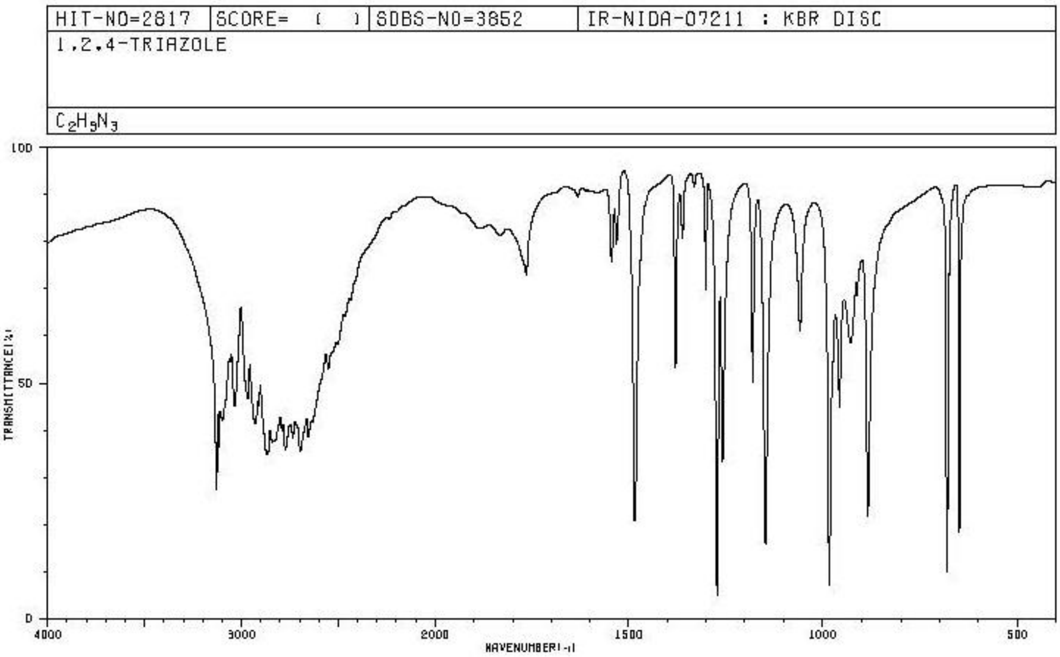

3129	26	2828	36	1832	79	1362	77	982	6
3097	41	2791	38	1765	70	1302	66	956	43
3034	43	2773	34	1833	86	1272	4	927	57
2967	44	2736	36	1644	72	1268	32	886	20
2930	39	2697	34	1532	77	1180	49	681	9
2866	33	2655	37	1485	20	1147	15	675	70
2840	36	2662	60	1380	60	1068	68	660	17

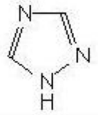

Supplementary Fig. 23. FT-IR spectrum of 1,2,4-triazole originated from Spectral Database for Organic Compounds SDBS. URL for this compound: https://sdbs.db.aist.go.jp/sdbs/cgi-bin/landingpage?sdbsno=3852.

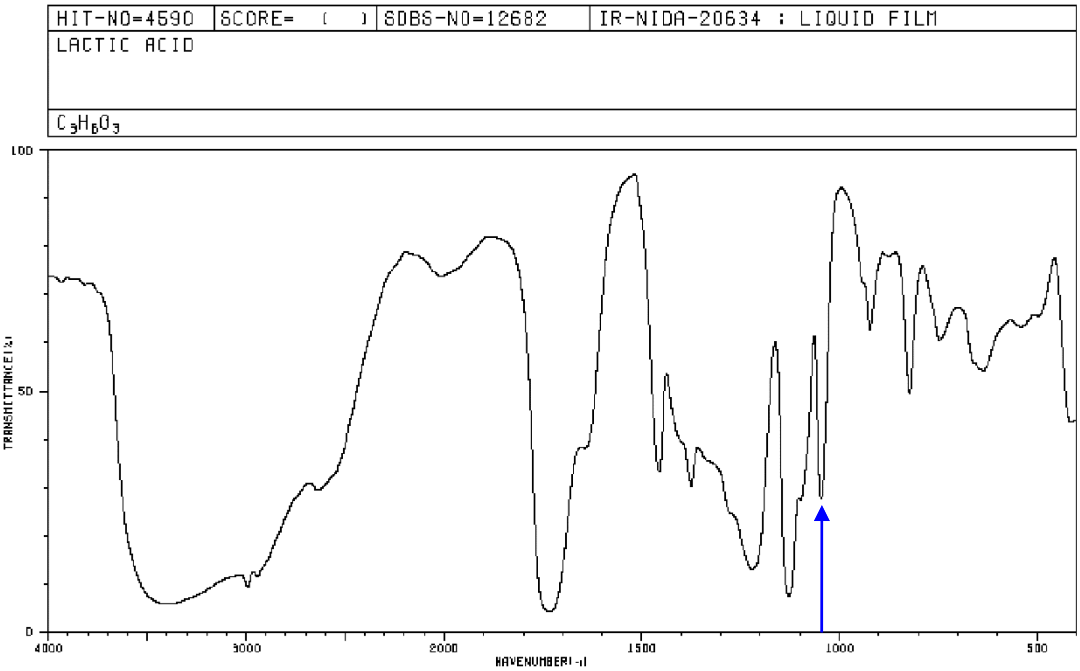

2990	10	1220	14	637	63
2943	12	1128	8	546	62
2833	30	1047	26	541	82
2010	72	923	62		
1732	4	823	50		
1456	34	747	60		
1376	31	642	63		

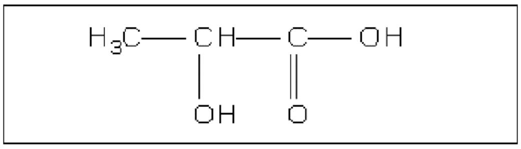

Supplementary Fig. 24. FT-IR spectrum of lactic acid originated from Spectral Database for Organic Compounds SDBS. The 1047 cm^{-1} peak is assigned to C–OH vibration. URL for this compound: http://sdbs.db.aist.go.jp/sdbs/cgi-bin/direct_frame_disp.cgi?sdbsno=12682.

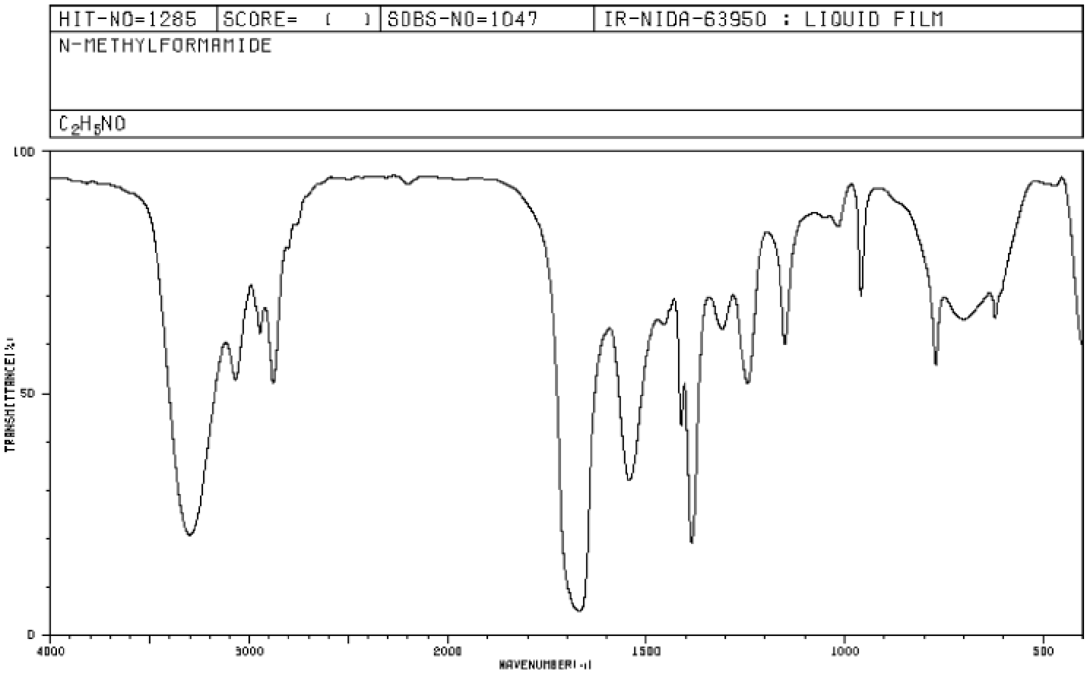

3298	20	1386	18	701	62
3067	50	1309	60	622	62
2945	80	1245	50		
2879	60	1161	68		
1668	4	1016	81		
1542	30	959	68		
1413	42	770	63		

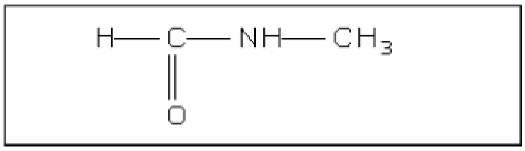

Supplementary Fig. 25. FT-IR spectrum of *N*-methylformamide [C_2H_5NO] originated from Spectral Database for Organic Compounds SDBS. URL for this compound: https://sdbs.db.aist.go.jp/sdbs/cgi-bin/direct_frame_top.cgi.

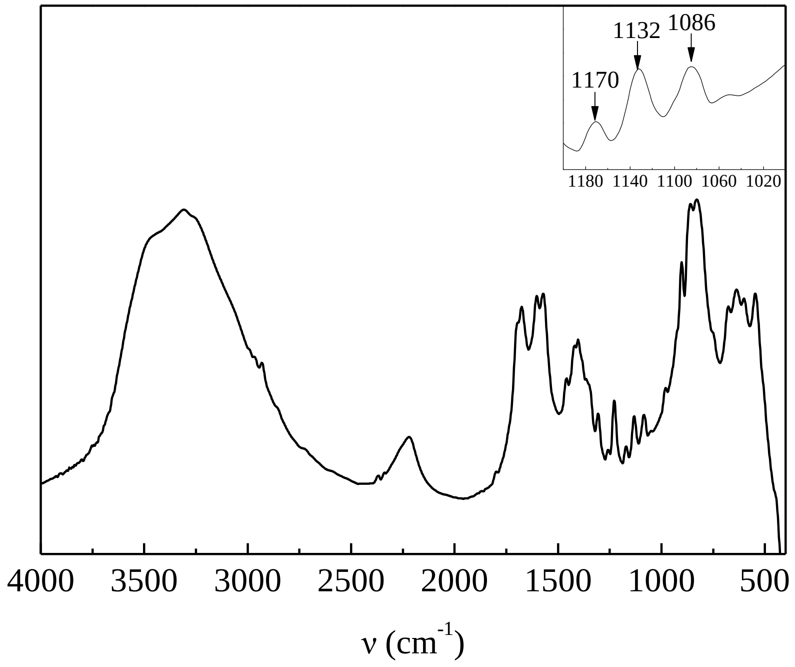

Supplementary Fig. 26. FT-IR spectrum of Na_3(Hhomocit)·H_2O.[7] The 1086 cm^{-1} peak is assigned to C–O_H vibration.

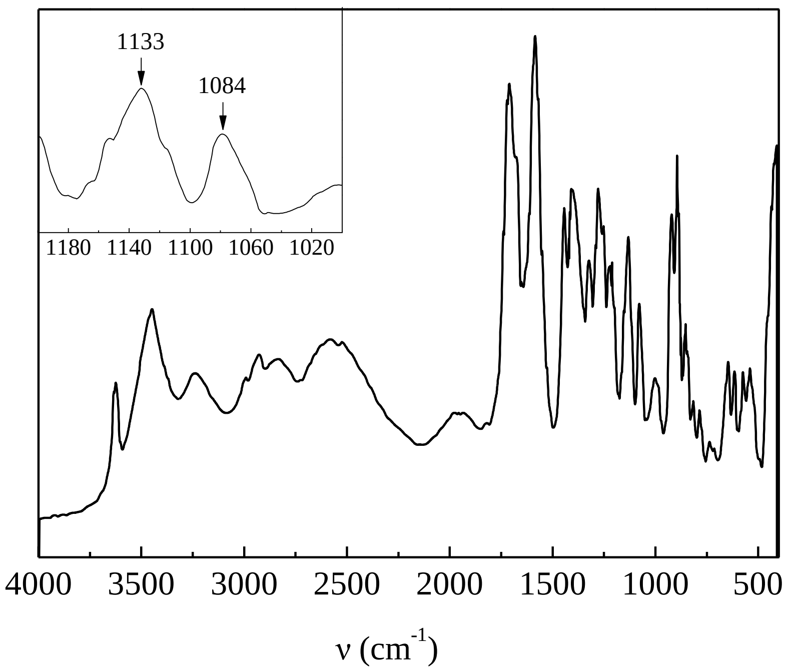

Supplementary Fig. 27. FT-IR spectrum of $K_2[Mo^{VI}O_2(R,S\text{-}H_2homocit)_2]\cdot 2H_2O$.[7] The peak at 1084 cm^{-1} is assigned to C–O vibration.

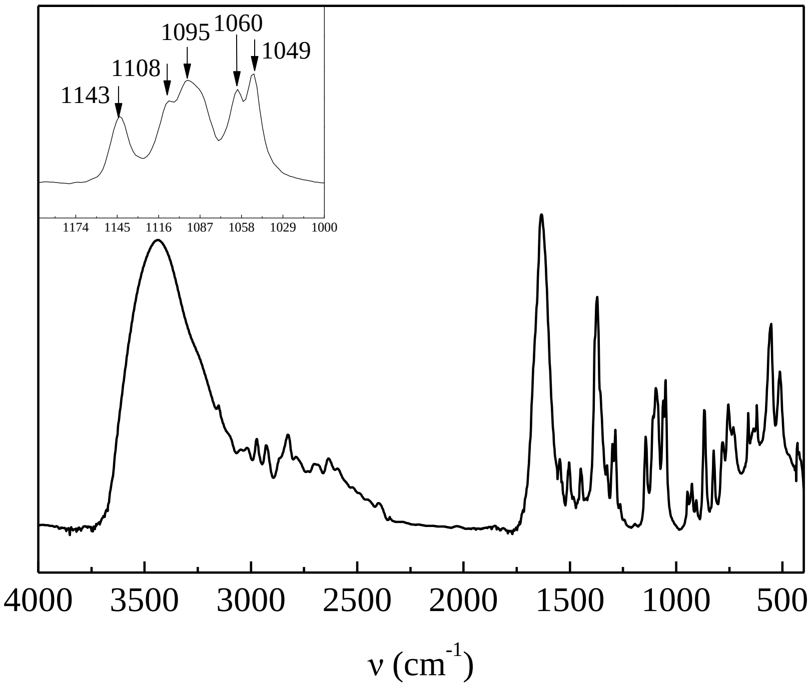

Supplementary Fig. 28. FT-IR spectrum of $Na_2[Mo_3SO_3(R,S\text{-lact})_3(im)_3]\cdot 10H_2O$.[5] The peaks at 1049, 1060 and 1056 cm^{-1} are assigned to C–O vibration.

Supplementary References

1. Modec B., Dolenc D. & Kasunič M. Complexation of molybdenum(V) with glycolic acid: an unusual orientation of glycolato ligand in $\{Mo_2O_4\}^{2+}$ Complexes. *Inorg. Chem.* **47**, 3625–3633 (2008).

2. Zhou Z. H., Hou S. Y., Cao Z. X., Wan H. L. & Ng S. W. Syntheses, crystal structures and biological relevance of glycolato and *S*-lactato molybdates. *J. Inorg. Biochem.* **98**, 1037–1044 (2004).

3. Wang S. Y., Dai J. W., Chen H. B. & Zhou Z. H. 2,2′-Bipyridine or 1,10-phenanthroline chelated oxomolybdenum(V) complexes with glycolate, lactate and malate in acidic media. *Inorg. Chim. Acta* **490**, 173–178 (2019).

4. Deng L., Dong X., An D. L., Weng W. Z. & Zhou Z. H. Gas adsorption of mixed-valence trinuclear oxothiomolybdenum glycolates. *Inorg. Chem.* **59**, 4874–4881 (2020).

5. Wang S. Y., Jin W. T., Chen H. B. & Zhou Z. H. Comparison of hydroxycarboxylato imidazole molybdenum(IV) complexes and nitrogenase protein structures: indirect evidence for the protonation of homocitrato FeMo-cofactors. *Dalton Trans.* **47**, 7412–7421 (2018).

6. Sokolov M. N. et al. Complexes of $M_3S_4^{4+}$ (M=Mo, W) with chiral alpha-hydroxy and aminoacids: synthesis, structure and solution studies. *Inorg. Chim. Acta* **395**, 11–18 (2013).

7. Chen C. Y. et al. Formations of mixed-valence oxovanadium^{V,IV} citrates and homocitrate with N-heterocycle chelated ligand. *Inorg. Chem.* **47**, 8714–8720 (2008).

8. Zhou Z. H., Hou S. Y., Cao Z. X., Tsai K. R. & Chow Y. L. Syntheses, spectroscopies and structures of molybdenum(VI) complexes with homocitrate. *Inorg. Chem.* **45**, 8447–8451 (2006).

9. Paju A., Kanger T., Pehk T., Eek M. & Lopp M. A short enantioselective synthesis of homocitric acid-γ-lactone and 4-hydroxy-homocitric acid-γ-lactones. *Tetrahedron* **60**, 9081–9084 (2004).

10. Sheldrick G. M. A short history of SHELX. *Acta Cryst.* **A64**, 112–122 (2008).

11. Dolomanov O. V., Bourhis L. J., Gildea R. J., Howard J. A. K. & Puschmann H. OLEX2: a complete structure solution, refinement and analysis program. *J. Appl. Cryst.* **42**, 339–341 (2009).

12. Sheldrick G. M. Crystal structure refinement with SHELXL. *Acta Cryst.* **C71**, 3–8 (2015).

13. Wang S. Y. & Zhou Z. H. Molybdenum imidazole citrate and bipyridine homocitrate in different oxidation states – balance between coordinated α-hydroxy

and α-alkoxy groups. *RSC Adv.* **9**, 519–528 (2019).

14. Zhou Z. H., Yan W. B., Wan H. L. & Tsai K. R. Synthesis and characterization of homochiral polymeric *S*-malato molybdate(VI): toward the potentially stereospecific formation and absolute configuration of iron-molybdenum cofactor in nitrogenase. *J. Inorg. Biochem.* **90**, 137–143 (2002).

15. Knobler C. B. et al. Molybdenum(VI) complexes with malic acid: their inter-relationships, and the crystal structure of dicaesium bis[(S)-malato($^{2-}$)]-cis-dioxomolybdate(VI)–water (1/1). *J. Chem. Soc. Dalton Trans.* **7**, 1299–1303 (1983).

16. Zhou Z. H., Wan H. L. & Tsai K. R. Syntheses and spectroscopic and structural characterization of molybdenum(VI) citrato monomeric raceme and dimer, $K_4[MoO_3(cit)]\cdot 2H_2O$ and $K_4[(MoO_2)_2O(Hcit)_2]\cdot 4H_2O$. *Inorg. Chem.* **39**, 59–64 (2000).

17. Zhang R. H. et al. Crystalline and solution chemistry of tetrameric and dimeric molybdenum(VI) citrato complexes. *Inorg. Chim. Acta* **406**, 27–36 (2013).

18. Zhou Z. H., Chen C. Y., Cao Z. X., Tsai K. R. & Chow Y. L. *N*-heterocycle chelated oxomolybdenum(VI and V) complexes with bidentate citrate. *Dalton Trans.* **252**, 2475–2479 (2008).

19. Zhou Z. H., Deng Y. F., Cao Z. X., Zhang R. H. & Chow Y. L. Dimeric dioxomolybdenum(VI) and oxomolybdenum(V) complexes with citrate at very low pH and neutral conditions. *Inorg. Chem.* **44**, 6912–6914 (2005).

20. Zhou Z. H., Wang H. X., Yu P., Olmstead M. M. & Cramer S. P. Structure and spectroscopy of a bidentate bis-homocitrate dioxo-molybdenum(VI) complex: Insights relevant to the structure and properties of the FeMo-cofactor in nitrogenase. *J. Inorg. Biochem.* **118**, 100–106 (2013).

21. Li D. M. et al. Synthesis and characterization of binuclear molybdenum–polycarboxylate complexes with sulfur bridges. *J. Inorg. Biochem.* **99**, 1602–1610 (2005).

22. Xing Y. H. et al. A new dinuclear molybdenum(V)-sulfur complex containing citrate ligand: synthesis and characterization of $K_{2.5}Na_2NH_4[Mo_2O_2S_2(cit)_2].5H_2O$. *Eur. J. Solid State Inorg. Chem.* **35**, 745–756 (1998).

23. Chen Q. L., Chen H. B., Cao Z. X. & Zhou Z. H. Synthesis, spectral, and structural characterizations of imidazole oxalato molybdenum(IV/V/VI) complexes. *Dalton Trans.* **42**, 1627–1636 (2013).

24. Brunauer S., Emmett P. H. & Teller E. Adsorption of gases in multimolecular layers. *J. Am. Chem. Soc.* **60**, 309–319 (1938).

25. Li Y. W. & Yang R. T. Gas adsorption and storage in metal−organic framework MOF-177. *Langmuir* **23**, 12937–12944 (2007).

26. Jin W. T. et al. Preliminary assignment of protonated and deprotonated homocitrates in extracted FeMo-cofactors by comparisons with molybdenum(IV) lactates and oxidovanadium glycolates. *Inorg. Chem.* **58**, 2523–2532 (2019).

27. Deacon G. B. & Phillips R. J. Relationships between the carbon-oxygen stretching frequencies of carboxylato complexes and the type of carboxylate coordination. *Coord. Chem. Rev.* **33**, 227–250 (1980).

28. Borchardt H. J. & Daniels F. Application of differential thermal analysis to the study of reaction kinetics. *J. Am. Chem. Soc.* **79**, 41–46 (1957).

29. Gowda A. S., Petersen J. L. & Milsmann C. Redox chemistry of bis(pyrrolyl)pyridine chromium and molybdenum complexes: an experimental and density functional theoretical study. *Inorg. Chem.* **57**, 1919–1934 (2018).

30. Mitra J. & Sarkar S. Oxo–Mo(IV)(dithiolene)thiolato complexes: analogue of reduced sulfite oxidase. *Inorg. Chem.* **52**, 3032–3042 (2013).

31. Ha V. T. T., Sarıoğlan A., Erdem-Şenatalar A. & Taârit Y. B. An EPR and NMR study on Mo/HZSM-5 catalysts for the aromatization of methane: Investigation of the location of the pentavalent molybdenum. *J. Mol. Catal. A: Chem.* **378**, 279–284 (2013).

32. Keable S. M. et al. Structural characterization of the P^{1+} intermediate state of the P-cluster of nitrogenase. *J. Biol. Chem.* **293**, 9629–9635 (2018).

33. Rutledge H. L. et al. Redox-dependent metastability of the nitrogenase P-cluster. *J. Am. Chem. Soc.* **141**, 10091–10098 (2019).

34. Henthorn J. T. et al. Localized electronic structure of nitrogenase FeMoco revealed by selenium k-edge high resolution x-ray absorption spectroscopy. *J. Am. Chem. Soc.* **141**, 13676–13688 (2019).
