# Supplementary material for: Assignment of protonated R-homocitrate in extracted FeMo-cofactor of nitrogenase via vibrational circular dichroism spectroscopy
Source: Commun Chem. 2020 Oct 28;3:145. doi: 10.1038/s42004-020-00392-z (PMC8323615; doi:10.1038/s42004-020-00392-z)
Supplement: Supplementary file 2 — Description of Additional Supplementary Files [file 42004_2020_392_MOESM2_ESM.pdf]

Description of Additional Supplementary Files

File Name: Supplementary Data 1
Description: Crystallographic data file in CIF format for 1

File Name: Supplementary Data 2
Description: Crystallographic data file in CIF format for 2

File Name: Supplementary Data 3
Description: Crystallographic data file in CIF format for 3
